# A confidence metric for using neurobiological feedback in actor-critic reinforcement learning based brain-machine interfaces

**DOI:** 10.3389/fnins.2014.00111

**Published:** 2014-05-26

**Authors:** Noeline W. Prins, Justin C. Sanchez, Abhishek Prasad

**Affiliations:** ^1^Department of Biomedical Engineering, University of MiamiCoral Gables, FL, USA; ^2^Department of Neuroscience, University of MiamiCoral Gables, FL, USA; ^3^Miami Project to Cure Paralysis, University of MiamiCoral Gables, FL, USA

**Keywords:** brain-machine interface, reinforcement learning, Hebbian, actor-critic, feedback

## Abstract

Brain-Machine Interfaces (BMIs) can be used to restore function in people living with paralysis. Current BMIs require extensive calibration that increase the set-up times and external inputs for decoder training that may be difficult to produce in paralyzed individuals. Both these factors have presented challenges in transitioning the technology from research environments to activities of daily living (ADL). For BMIs to be seamlessly used in ADL, these issues should be handled with minimal external input thus reducing the need for a technician/caregiver to calibrate the system. Reinforcement Learning (RL) based BMIs are a good tool to be used when there is no external training signal and can provide an adaptive modality to train BMI decoders. However, RL based BMIs are sensitive to the feedback provided to adapt the BMI. In actor-critic BMIs, this feedback is provided by the critic and the overall system performance is limited by the critic accuracy. In this work, we developed an adaptive BMI that could handle inaccuracies in the critic feedback in an effort to produce more accurate RL based BMIs. We developed a confidence measure, which indicated how appropriate the feedback is for updating the decoding parameters of the actor. The results show that with the new update formulation, the critic accuracy is no longer a limiting factor for the overall performance. We tested and validated the system onthree different data sets: synthetic data generated by an Izhikevich neural spiking model, synthetic data with a Gaussian noise distribution, and data collected from a non-human primate engaged in a reaching task. All results indicated that the system with the critic confidence built in always outperformed the system without the critic confidence. Results of this study suggest the potential application of the technique in developing an autonomous BMI that does not need an external signal for training or extensive calibration.

## Introduction

In recent years, Brain-Machine Interfaces (BMIs) have been shown to restore movement to people living with paralysis via control of external devices such as computer cursors (Wolpaw and McFarland, 2004; Simeral et al., 2011), robotic arms (Hochberg et al., 2006, 2012; Collinger et al., 2013), or one's own limbs through functional electrode stimulation (FES) (Moritz et al., 2008; Pohlmeyer et al., 2009; Ethier et al., 2012). Studies have shown that the BMI control can be affected by several factors such as the type of neural signals used (Wessberg et al., 2000; Mehring et al., 2003; Andersen et al., 2004; Sanchez et al., 2004), long-term stability of the input signals (Santhanam et al., 2006; Flint et al., 2013), type of training signals used for decoders (Miller and Weber, 2011), type of decoders (linear, non-linear, static, adaptive) (Kim et al., 2006; Shenoy et al., 2006; Bashashati et al., 2007; Li et al., 2011), and cortical plasticity that occurs during BMI use (Sanes and Donoghue, 2000; Birbaumer and Cohen, 2007; Daly and Wolpaw, 2008). Other factors include the type of signal used [local field potentials (LFPs), electrocorticograms (ECoG), single or multiunit activity] and the long-term stability of the signals (Schwartz et al., 2006; Chestek et al., 2011; Prasad et al., 2012). Additionally, the performance can also be affected by perturbations such as loss or gain of neurons, noise in the system, electrode failure, and changes in the neuronal firing characteristics (Maynard et al., 1997; Shoham et al., 2005; Patil and Turner, 2008; Pohlmeyer et al., 2014). These factors occur dynamically in nature and affect long-term BMI performance. Therefore, there is a need to produce more stable, high performance BMIs that are less affected by these daily changes in the neural input space due to the above interactions so that they can be reliably implemented in activities of daily living (ADL).

Traditionally, BMIs utilize a decoder that translates neural signals into executable actions by finding the mapping between the neural activity and output commands. Due to the non-stationarity of the neural data (Snider and Bonds, 1998), many of these decoders need to adapt its parameters in order to find an optimal mapping between the neural control signals and the output motor actions. Commonly used decoders (such as Wiener models and Kalman filters) are trained using supervised learning (SL) techniques that require a training data set and a desired output value, which is usually a real or inferred kinematic signal from a limb (Schalk et al., 2007; Gilja et al., 2012). However, this paradigm poses challenges for paralyzed individuals who may not be able to generate a training kinematic signal in order to create a stable mapping between the motor control signals to BMI command outputs. Maladaptive cortical reorganization occurring due to non-use of the paralyzed limbs further worsens the reliable extraction of training kinematic signals in such individuals (Elbert and Rockstroh, 2004; Di Pino et al., 2012). Studies have used motor imagery, baseline neural activity, random initialization of the decoder, and ipsilateral limb movements to create training signals that can be used to initialize the BMI decoder and then refine the decoder during the experiment (Pfurtscheller and Neuper, 2001; Bai et al., 2010). All these approaches are based on the SL paradigm where the presence of an external training signal is critical to achieve optimal BMI control and requires initial time-consuming calibration (which can range from 10 min to about an hour) of the BMI decoder before each session to adapt to the perturbations in the neural environment.

Unsupervised learning (UL) techniques provide an alternative to SL models as they only rely on the structure of the input data and finds patterns within the data itself (Shenoy and Rao, 2005; Rao, 2010; Vidaurre et al., 2011; Gürel and Mehring, 2012). This is particularly useful for BMI applications where the user may not be able to generate reliable kinematic signals and the input signals are affected by the changing dynamics of the neural environment. However, if the input space changes in an unpredictable manner or there are perturbations present unsupervised decoders may not be mapped appropriately to the behavior since they rely on the structure of the training data. For example, *k*-means, an unsupervised clustering method uses the structure of the training data to define clusters. When the statistics of the data change between training and testing, an optimal solution is not guaranteed (Fisher and Principe, 1996; Snider and Bonds, 1998; Antoni and Randall, 2004). Therefore, in order to address these challenges we have utilized a semi-supervised learning technique based on Reinforcement Learning (RL), which depends on performance outcomes and not on explicit training signals (Sutton and Barto, 1998). In comparison to SL techniques, RL uses an instantaneous feedback to modify its parameters but does not require an explicit training signal. Since there is a structure already present (due to its feedback) RL is able to respond to perturbations better than UL. The basic idea of RL is for an “agent” to make actions on an “environment” and receive an instantaneous “reward” in order to maximize the cumulative or long term reward the “agent” receives. In this case, the “agent” is an intelligent system (e.g., BMI decoder), which selects an action out of many available actions with an aim to maximize the long-term reward. An action will change the state of the environment (action space) from one state to another, for example, move left or move up. The “reward” is the evaluation of the action selected depending upon its outcome. A good outcome will lead to a high reward and vice versa.

Theoretical models of learning have been developed for different brain areas which suggest that the cerebellum, the basal ganglia, and the cerebral cortex are specialized for different types of learning (Houk and Wise, 1995). SL, based on an error signal has been proposed to be handled by the cerebellum, while the cerebral cortex is specialized for UL and the basal ganglia are specialized for RL based on the reward signal (Doya, 2000). We used a particular class of RL known as the actor-critic RL in this study, which provides us with a framework to obtain the reward feedback from a different source than that of the action. The “actor” makes decisions of which action to choose from, while the “critic” gives feedback on the appropriateness of this decision. In other words, the critic criticizes the choice made by the actor. In contrast to SL decoders, RL does not need an explicit training signal. RL also gives a framework for adding more biological realism into the structure of the decoder design. We have shown earlier an actor-critic RL as a framework for using an evaluative feedback in neuroprosthetic devices (Mahmoudi and Sanchez, 2011). This framework provides a structure where a user and the agent can both co-exist and work toward a common goal. We have also shown how convergence, generalization, accuracy and perturbations take place in a Hebbian RL framework (Mahmoudi et al., 2013) and that adaptation is necessary for maintaining BMI performance following neural perturbations (Pohlmeyer et al., 2014). In these studies, the actor was driven by the motor neural data and the critic feedback was computed by comparing the action taken to the desired action. The drive is to move toward an autonomous BMI which does not need to know the desired action and would not need an external training signal of any kind. Therefore, to bring biological realism for building a fully autonomous BMI system, we have investigated the possibility of using a reward signal from the brain itself to drive the critic (Prins et al., 2013). There are multiple reward areas in the brain, which can be used to extract such information such as the striatum (Phillips, 1984; Wise and Bozarth, 1984; Wise and Rompré, 1989; Schultz et al., 1992, 2000; Tanaka et al., 2004), cingulate (Shima and Tanji, 1998; Bush et al., 2002; Shidara and Richmond, 2002), and orbitofrontal cortices (Rolls, 2000; Schultz et al., 2000; Tremblay and Schultz, 2000); most notably the striatum that is involved in the perception action reward cycle (PARC) (Apicella et al., 1991; Pennartz et al., 1994; Hollerman et al., 1998; Kelley, 2004; Nicola, 2007), which is the circular flow of information from the environment to sensory and motor structures and back again to the environment completing the cycle during the processing of goal-directed behavior. All adaptive behaviors require the PARC and the control of goal-directed actions relies on the operation of such an information-movement cycle. A critic driven by such a biological source (biological critic) would not only be mimicking a biological system and adding more biological realism, but also render toward an autonomous BMI which does not need a training signal; however, the challenge is how to incorporate a biological critic in to this actor-critic RL framework to maximize the BMI performance. We have found from preliminary analysis that the reward signals and reward representations are diverse and leads to lower accuracy when classified. This is due to the finding that the overall performance of the decoder model is limited by the critic accuracy (Pohlmeyer et al., 2014). This occurs because updating the system with wrong feedback perturbs the temporal sequence of the RL trajectory and can lead to a suboptimal decoding solution. When the critic feedback is less than perfect, the actor is only able to achieve an accuracy with the critic accuracy as its upper limit (Pohlmeyer et al., 2014). Therefore, there is a need to develop a framework that can handle inaccuracies due to uncertainty in the critic feedback so that a biological critic can be used to drive an autonomous BMI.

In this study, we developed a novel method for decoupling the overall performance from the accuracy of the critic by adding a confidence measure in the critic feedback. Using this method, the system only updates when the critic is accurate. The accuracy can be derived from the distance to the boundary for the decision surface for rewarding and non-rewarding actions. We performed simulations for this novel method on both synthetic and non-human primate (NHP) data to show that the overall performance can be increased above the critic accuracy to create high performance BMIs. We used a two-choice task to show proof of concept that a system with built-in confidence measure is able to perform significantly better than a system without the confidence measure. Such a system can be expanded to complex tasks that include a larger number of targets where the critic output is still in the form of two states similar to one shown in this study (Mahmoudi et al., 2013). This new method of confidence driven updates is particularly effective when the accuracy of the biological critic is low.

## Methods

### Hebbian reinforcement learning

**Figure 1:**
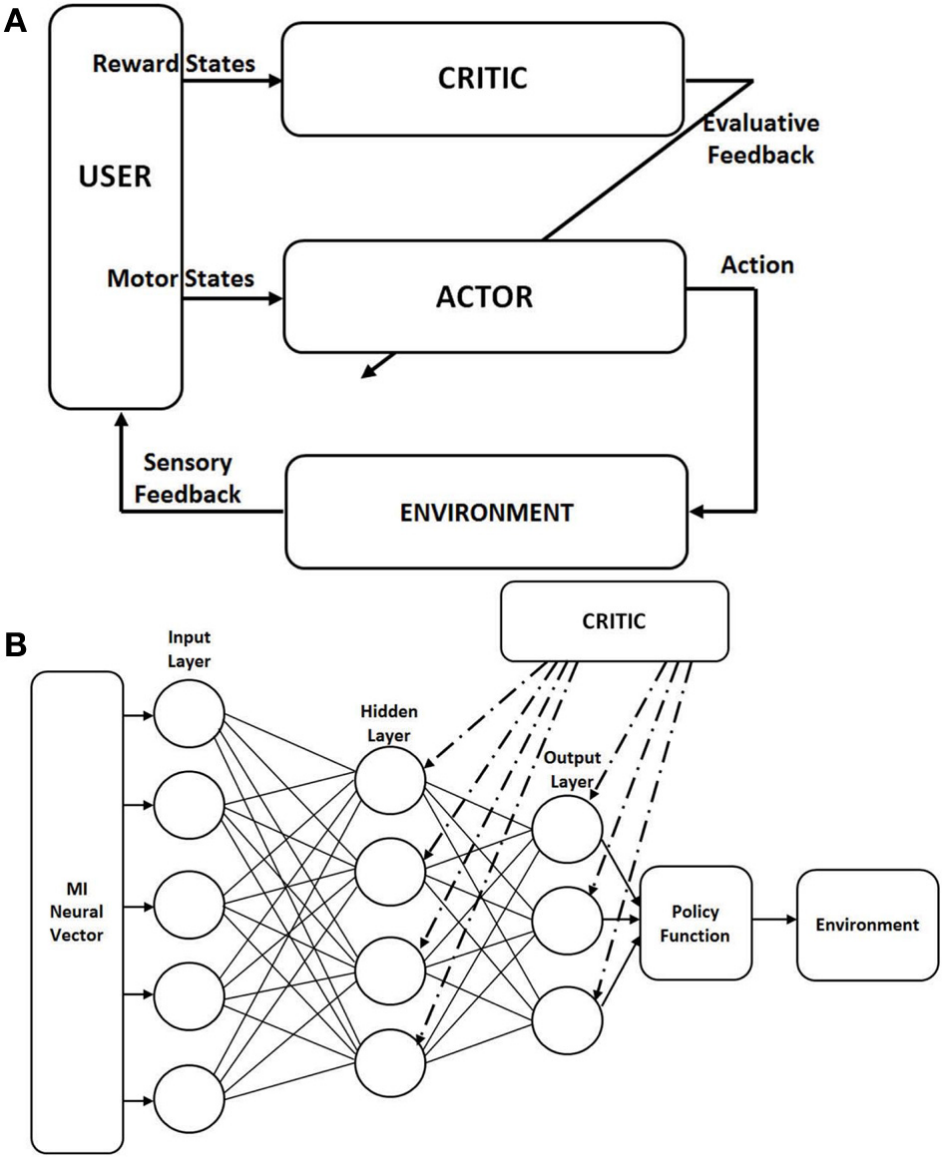
**Architecture of the actor-critic reinforcement learning (RL). (A)** Classical actor-critic RL architecture as adapted for Brain-Machine Interface (BMI). The actor maps the neural commands into actions to control the external device. The actor is driven by the motor neural commands. The critic gives an evaluative feedback about the action taken based on its reward. This evaluative feedback is used to update the weights of the actor. The critic is driven by the neural data from the striatum for an autonomous BMI. **(B)** Actor network structure in the actor-critic RL; fully connected feed forward neural network with binary nodes, with 5 nodes in the hidden layer. The policy function used is the “greedy” policy which selects the node with the highest value at the output layer and channels that action to the environment. The critic gives an evaluative feedback to all nodes in the output and hidden layers. This modulates the synaptic weight updates based on the local pre- and postsynaptic activity.

We used the actor-critic RL paradigm to test our decoder in which the BMI decoder that decodes the action is embedded within the actor architecture itself. We modified the weight updates according to the Hebbian rule, called the Hebbian Reinforcement Learning (HRL) (Pennartz, 1997). RL learns by interaction to map neural data to output actions in order to maximize the cumulative reward. For this, there are two functions: the value and policy functions. The value function provides the reward value and the policy function provides a method of choosing from a variety of available actions. In actor-critic RL, the structure is such that the policy is independent of the value function. The policy is given by the “actor” and the value function is given by the “critic” (Sutton and Barto, 1998). The actor chooses which action to execute out of the many actions possible and the parameters of the actor is changed according to the evaluative feedback given by the critic (Figure 1A).

The Hebbian learning rule specifies how much the weights between two neurons must be changed in proportion to their activation (Pennartz, 1997; Bosman et al., 2004). HRL is a class of associative RL where the local presynaptic and postsynaptic activity in the network is correlated with a global reinforcement signal (Gullapalli, 1991; Kaelbling, 1994). Figure 1B shows the network structure we are using for our model where the actor is an artificial neural network (ANN) with 3 layers. The input layer receives motor neural data and the output layer gives the value for each action available. Each processing node in the output layer represents one possible action. The policy we are using is the “greedy policy,” which says that the action with the highest value is chosen and implemented. Each node in the hidden and output layers is a processing element (PE). Each of these PE has Equation 2 in its entirety which is known as the associative reward-penalty algorithm in adaptive control theory (Barto and Anandan, 1985). The input to each PE is *x*_*i*_ (firing rate of the neuron *i* in a given bin) and the output is *x*_*j*_. For the output node *j*, with the transfer function *f*(·), *x*_*j*_ is given by

(1) $$x_{j}=sgn\left[P_{j}\right]=sgn\text{​}\left[f\left(\sum_{i}w_{ij}x_{i}\right)\right]$$

Where $P_{j}=f\left(\sum_{i}w_{ij}x_{i}\right)$. We have used a hyperbolic tangent as the transfer function. The weight update rule for HRL is given by:

(2) $$\Delta \omega_{ij}=\mu^{+}r\left(x_{j}-P_{j}\right)x_{i}+\mu^{-}\left(1-r\right)(1-x_{j}-P_{j})x_{i}$$

where the reward, r evaluates the "appropriateness" of the PE's output (−1 ≤ *r* ≤ 1), *x*_*j*_, due to the input *x*_*i*_. μ^+^ and μ^−^ represent the learning rates for the reward and penalty components, respectively (Mahmoudi et al., 2013). The first term corresponds to the reward and the second term corresponds to the penalty. There are two unique cases for this equation. The first case is when *r* = 1, there is contribution only from the first term and the weight update equation (Equation 2) becomes:

(3) $$\Delta \omega_{ij}=\mu^{+}r\left(x_{j}-P_{j}\right)x_{i}$$

This means that in rewarding trials (*r* = 1), only the positive component contributes to the weight update. But in non-rewarding trials (*r* = −1), both terms contribute and the system is more sensitive to the negative feedback. The second case is when *P*_*j*_ approaches *x*_*j*_ there is contribution only from the second term, hence the weight update becomes:

(4) $$\Delta \omega_{ij}=\mu^{-}\left(1-r\right)(1-x_{j}-P_{j})x_{i}$$

In this case, the system will only adapt for negative feedback. When both the above conditions are achieved, (*r* = 1 and *P*_*j*_ → *x*_*j*_), the weights will not update further. During instances where there is no weight update, the system has consolidated the functional relationship between input and output. Unless and until there is a negative feedback, the system will not update further.

### Critic confidence

The decoder in the actor incorporated a confidence measure that indicated the accuracy of the critic. This was motivated by our previous findings that the overall performance of the system was affected by the critic accuracy (Pohlmeyer et al., 2014) and that the accuracy of extracting reward signal from the neural data was less than 90% (Prins et al., 2013). The formulation adds an additional term in the HRL weight update equation (Equation 2), which indicated how much confidence the critic had in the feedback value. We defined this term as the confidence (ρ) and hence, the modified HRL weight update equation (Equation 2) becomes:

(5) $$\Delta \omega_{ij}=\mu^{+}\rho r\left(x_{j}-P_{j}\right)x_{i}+\mu^{-}\left(1-\rho r\right)(1-x_{j}-P_{j})x_{i}$$

where ρ is the confidence in the feedback, *r*. Here, the critic determines the appropriateness of the action taken by the actor. The critic gives an output of ±1 (*r* = ±1) indicating if it was an action to be rewarded or penalized. In addition, the critic also gives a value of the confidence (ρ) it has on the feedback given. If the confidence is high, the actor is updated but if it is low, the actor is not updated. This is to be determined by the value of ρ given by the critic. Depending on the confidence given after each action is taken, the actor weights are updated only when the critic confidence is high. Since noise in feedback data can tend to add uncertainty closer to the decision boundary, more noisy data can result in lower levels of confidence and the actor weights are not updated as frequently. This system however, does not address the problem of mislabeled critic trials (i.e., wrong feedback with high confidence). By not updating (i.e., not changing the weights) when the confidence in critic feedback is low, it provides a mechanism for preventing inaccuracies from entering into the system. The trade-off for this approach is that the number of samples needed to train the system can be more since every sample may not be used if the confidence is low.

In the simulations, we varied the critic accuracy from 50 to 100%. An N% accurate critic means that (1-N)% of the time it will be incorrect. The actor is blind to N, but for these simulations we provided boolean confidence information to the actor (ρ = {0, 1}). Thus, in these simulations, the actor with confidence does not know how accurate the critic is, but knows exactly when the critic provided accurate feedback. This actor does not adapt at all if the feedback was inaccurate (i.e., ρ = 0). In contrast, the standard actor (without confidence) adapts fully to both the accurate and inaccurate feedback.

### Generating neural data

We generated synthetic neural data and tested it on the HRL update equation both without (Equation 2) and with confidence (Equation 5) to compare the system performance. The performance in each session was quantified by the number of correct actions for that particular session. For synthetic data, one session was considered as one simulation and each session consisted of 100 trials (actions). We also included additional noise by changing the stimulus (how the synaptic current, *I*, is generated in Equation 6). For each different set of *I*, we generated data, performed the simulations and tested the performance. Finally, we tested the robustness of the model by using neural data from a NHP performing a two choice reaching task and compared performance. For the NHP data, one simulation consisted of 97 trials collected over 2 consecutive days. The results presented are a mean of 1000 simulations for both synthetic and NHP data.

#### Generating MI synthetic data for the actor

The synthetic neural data used to test the model was generated by the standard Izhikevich method (Izhikevich, 2003) where the model was given by

(6) $$v^{\prime }=0.04v^{2}+5v+140-u+I$$

(7) $$u^{\prime }=a\left(bv-u\right)$$

with the auxiliary after-spike resetting

(8) $$\;if\;v\geq +30mV\;then\;\{\begin{array}{c} v\leftarrow c\;\\ u\leftarrow u+d \end{array}$$

Here *v* was the membrane potential of the neuron and *u* represents a membrane recovery variable, which accounted for the activation/inactivation of ionic currents, and it provided negative feedback to *v*. After the spike reached its apex (+30 mV), the membrane voltage and the recovery variable were reset. The synaptic current is given by the variable, *I*, which was calculated from the stimulus of “1” for spike and “0” at all other times. For excitatory cells, *a* = 0.02, *b* = 0.2, (*c*, *d*) = (−65, 8) + (15, −6) · *e*^2^ where *e* is a random variable uniformly distributed, *e* ∈ [0, 1] (Izhikevich, 2003). We generated two motor states (motor state 1 and motor state 2) using the above model to depict two actions. The neural data was generated in 3 ensembles, one ensemble each tuned to one state (activity of the particular ensemble correlated with one state) and the third ensemble not tuned to either state simulating noise in real neural data.

#### Neural perturbations—additional noise in data

While the synthetic data was generated using a biologically realistic model, there are dynamic factors, which contribute to forms of noise not considered in the model. These are factors such as neurons dropping, electrodes deteriorating or breaking and encapsulation. Without making the model more complicated to mimic the noisy physiological system, we introduced additional noise to the synthetic data by adding a probability component to the stimulus, which generated the *I* in Equation 6. The actual value of noise in the stimulus was decided by a Gaussian distribution instead of the “1” or “0” as before. The number of neurons with this additional noise was varied from 0 to 100% in 10% increments. This additional probability component resulted in overlapping classes; the higher the probability component, more overlapping in the states generated. This was verified graphically using the first two principal components and confirmed that as the probability component to generate *I* was increased, the overlapping of the two classes also increased.

#### Simulations using NHP data

To validate our simulation results, a two choice decision making task was designed and neural signals were acquired while the monkey performed the task. We varied the critic accuracy from 50 to 100% in 10% increments and evaluated the performance. The experiments were conducted by a marmoset monkey (Callithrix jacchus) implanted with a 16 channel microwire array (Tucker Davis Technologies (TDT), Alachua, FL) targeting the hand and arm region in the primary motor cortex (MI). Neural data was acquired at 24,414.06 Hz using a TDT RZ2 system and bandpass filtered 300–5000 Hz. Thresholds were set manually by the experimenter and 20 multi-unit signals were isolated in real-time based on waveform and amplitude of the isolated waveforms. We did not distinguish between single unit and multi-unit activity. All the procedures were consistent with the National Research Council Guide for the Care and Use of Laboratory Animals and were approved by the University of Miami Institutional Animal Care and Use Committee.

**Figure 2:**
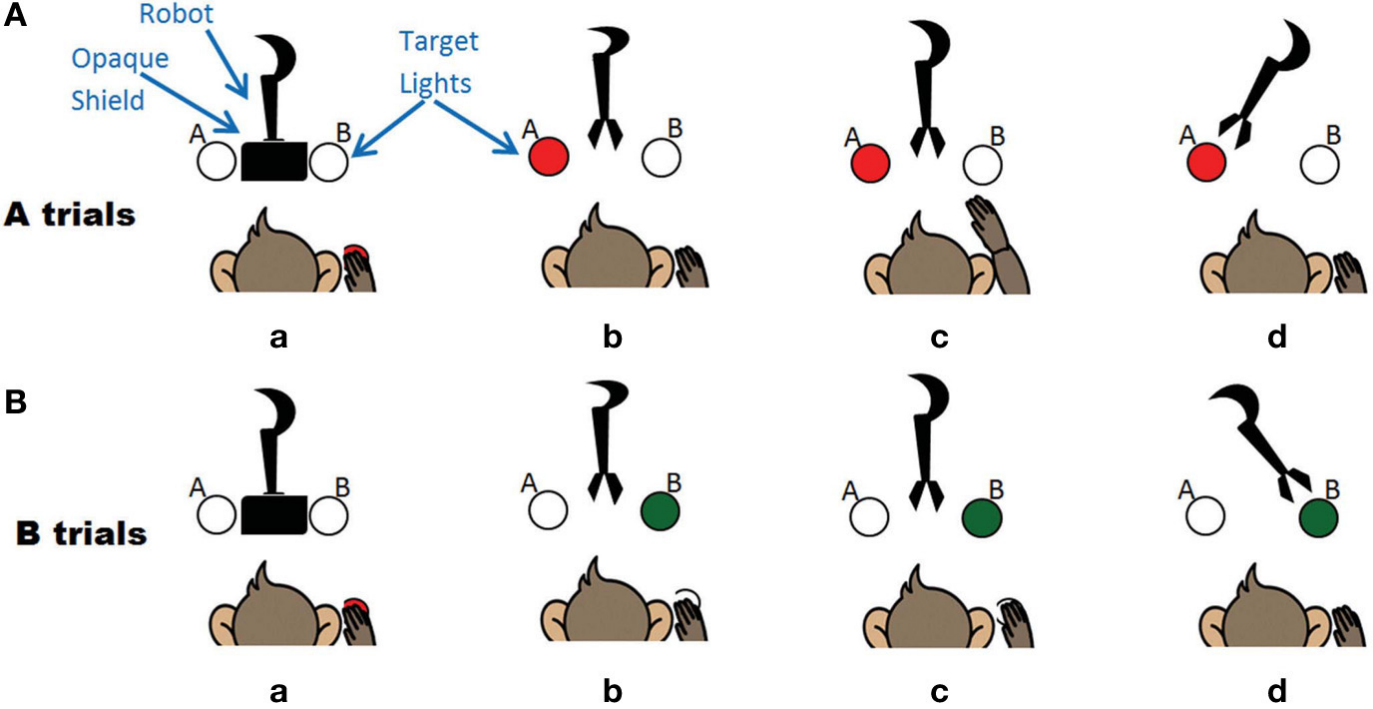
**The experiment where the monkey controls the robot arm. (A)** A trials associated with a motor high and the left target. Sequence of events (a) monkey triggers trial (b) Robot moves out from opaque screen, target A lights up (c) Monkey makes arm movement (d) Robot moves to target A. **(B)** B trials associated with a motor low and the right target. Sequence of events (a) monkey triggers trial (b) Robot moves out from opaque screen, target B lights up (c) Monkey keeps hand still (d) Robot moves to target B.

The task was a two-choice decision making task where the monkey was trained to move a robot arm to one of two targets to receive a food reward (Figure 2). A trial was initiated by the monkey when he placed his hand on a touchpad for a random (700–1200 ms) hold period. The trial onset was an audio cue that corresponded to a robot arm moving upwards from behind an opaque shield and presenting its gripper in front of the animal. The gripper held either a desirable (waxworm or marshmallow, “A” trials) or undesirable (wooden bead, “B” trials) object. Simultaneously, the A (red) or B (green) spatial target LED corresponding to the type of object in the gripper was illuminated. For A trials, the monkey had a 2 s window to reach to a second sensor to move the robot to A, while for B trials, he was required to keep his hand still on the touchpad for 2.5 s and the robot would move to B target. If the robot moved to the target illuminated, for both A and B trials, the monkey received a food reward. If the animal either did not interact with the task or performed the wrong action, these trials were removed from the analysis. The firing rate over a 2 s window following the trial start cue was used as input to the decoder.

## Results

We tested the model using 3 different data sets in one-step (classification) mode. Data sets used were: (1) synthetic data generated by an Izhikevich neural spiking model, (2) synthetic data with a Gaussian noise distribution, and (3) data collected from a non-human primate engaged in a reaching task. We varied the critic accuracy from 50 to 100% and ran two sets of simulations (S1 and S2) for each of the three data sets; S1, updated the actor at every trial and S2 updated only when the critic feedback was correct (i.e., confidence high). This was performed to compare whether it was better to adapt after each trial or only when the critic feedback was correct. For the purpose of these simulations, we used the correct critic feedback to indicate a high confidence of “1” and an incorrect critic feedback to indicate a low confidence of “0.” This can be determined empirically by the critic data that would require an in-depth evaluation, which was not the focus of this study. Since the decoder started at a naïve state, we used a pseudo-real time normalizing of the inputs before feeding to the network. This prevented any bias due to the difference in the magnitude of the inputs. This was done by keeping a real time record of the highest firing rate detected for each input, and then used to continually update the normalization parameters throughout the session (Pohlmeyer et al., 2014).

### Comparison of actor's performance with and without confidence measure

**Figure 3:**
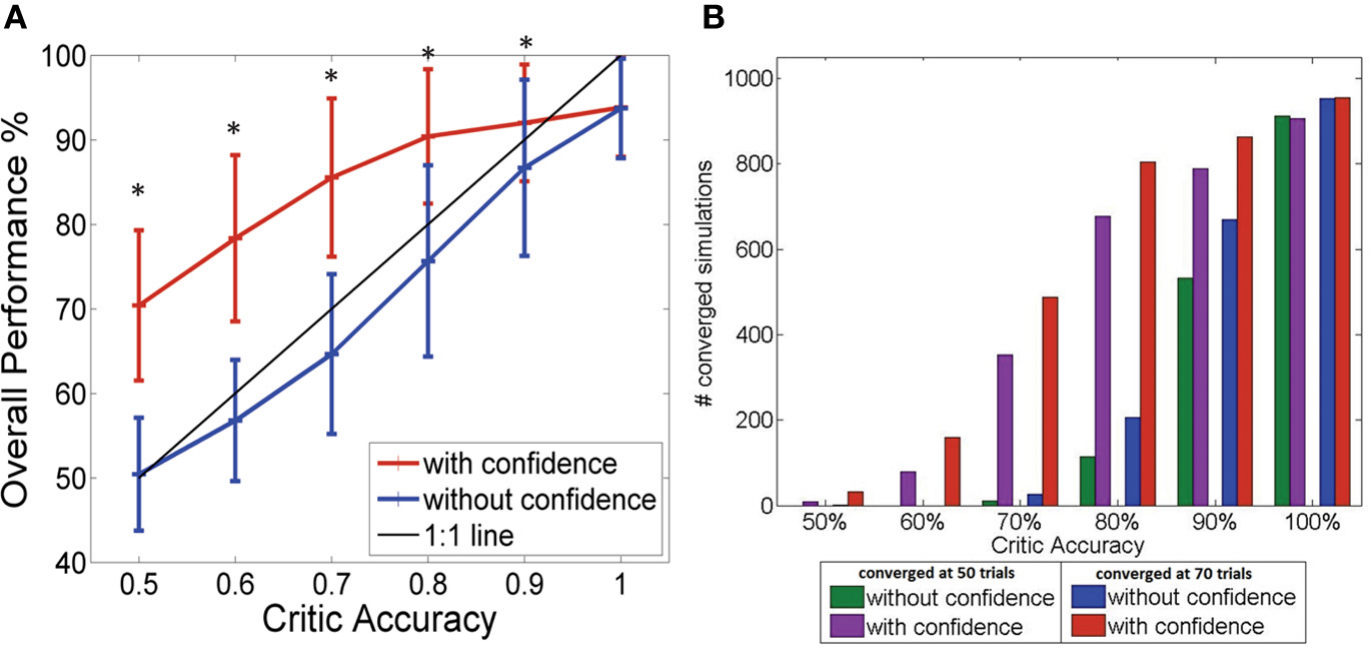
**(A)** Performance of the BMI Vs the critic accuracy with and without confidence inbuilt. (mean ± standard deviation. One thousand simulations. One hundred trials per simulation). Red: New update rule with confidence. Blue: Previous method with no confidence. Black: 1:1 relationship. Critic accuracy was varied from 50 to 100% with 100% being the best. ^*^Shows the values which showed statistical significant difference (alpha 0.001). The overall performance of the blue curve is limited by the accuracy of the critic but the overall performance of the red curve is able to go beyond the critic accuracy, decoupling the performance from the critic accuracy. **(B)** Stability of the system without (green/blue) and with (purple/red) confidence. Plot shows the number of simulations that maintained 100% accuracy beyond 50 trials (green/purple) and beyond 70 trials (blue/red).

Figure 3A shows how the performance level increased as the critic accuracy increased. The actor which was updated every time is shown in blue. The performance was always below the 1:1 curve showing how the actor performance is limited by the critic accuracy. However, the performance of the system where the actor was updated only when the critic was confident (shown in red) was able to perform above the critic accuracy level as seen in the figure. The performance increased from 50% (±6.6%) to 70% (±8.8%) at critic accuracy of 50% and further improved from 87% (± 10.4%) to 92% (±6.9%) at critic accuracy of 90%. A critic accuracy of 90% means that the critic gave a correct feedback 90% of the trials and wrong feedback 10% of the trials. For example, in our simulations each consisting of 100 trials, a 70% accurate critic gave correct feedback in 70 trials and wrong feedback in 30 trials. If there was no confidence built-in, the actor assumes that the value was always correct. In this new system with confidence built in, we reduced the confidence of the wrong feedback to zero. At lower critic accuracies (50, 60, and 70%), the system with the confidence outperformed the system without the confidence by approximately 20%. The performance of the two systems showed significant difference for all critic accuracy levels from 50 to 90% (Student's paired *t*-Test, with a two-tailed distribution, alpha 0.001—shown with ∗ in the figure). By updating weights accurately, the system learned optimal mapping and stabilized with time. Given that the system began with random initial conditions, there was no guarantee that the system would stabilize. Figure 3B gives a summary of the number of simulations out of 1000 that stabilized after 50 trials and 70 trials with and without the confidence. The convergence or stability was defined as maintaining 100% accuracy (last 50 trials or last 30 trials). The number of simulations that did stabilize at lower critic accuracies was higher for the system with the confidence measure. At higher critic accuracy levels, the overall performance was no longer limited by the critic accuracy but by the data itself. As the critic confidence increased, the difference in performance between the two systems became smaller and converged to a single value (94 ± 5.8%) since at 100% critic accuracy, both systems effectively have the same update equation.

**Figure 4:**
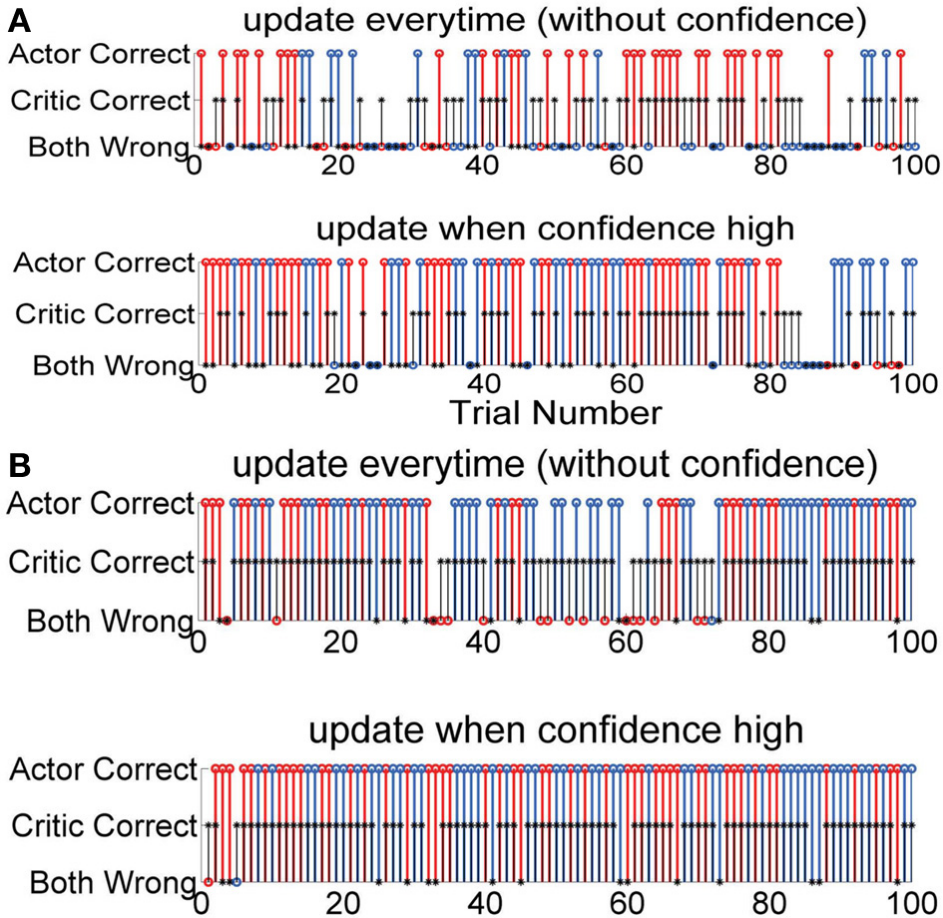
**Performance of each decoder during the length of the experiment for one simulation starting at random initial conditions**. One hundred trials. Red: Action 1, Blue: Action 2, Black: Critic. **(A)** Critic accuracy 60%. Both decoders perform correctly in the first trial but the critic gives a wrong feedback. The first system changes the weights causing the second trial to be wrong. Again, the critic gives a wrong feedback causing the third trial also to be wrong. Since the system weights are updated every time, wrong critic feedback causes the system to perform below the critic accuracy. However, in contrast even though the second subplot also starts the first trial the same way, the erroneous feedback does not affect it and the decoder is able to perform better than the first system. **(B)** Critic accuracy 80%. The first system starts with a correct action, but is very sensitive to wrong critic feedback. The second system starts with a wrong action, but by the 6th trial is able to achieve good performance and maintain throughout the rest of the session.

Figure 4 shows the details of the action selected in each trial and also the critic values for that particular trial. Figure 4A has two sets of simulations S1 and S2 and Figure 4B also has two sets of simulations S1 and S2. Each simulation started with random initial conditions. Figures 4A,B shows two such examples with two different critic accuracy levels. The critic accuracy was changed randomly based on the percentage given to the decoder. In Figure 4A, the critic is 60% accurate and the top subplot shows the performance of the system if the actor was updated every time (S1). The overall performance in this case is 47%. The first trial was correct, but the critic gave a wrong feedback and the actor weights were updated with this erroneous feedback causing the second trial to be wrong. When the critic gave a correct feedback during the third trial, the system started performing correctly. However, due to the erroneous feedback the performance was not stable. Even when the actor chose the correct action, if the critic provided a wrong feedback, it decreased the performance. In contrast, the second subplot shows the performance when the actor was updated with a confidence level (S2). For the same neural data, order of trials and critic feedback, the performance of the second system is 80%. Even though the critic gave wrong feedback at first, the actor learned to ignore this and was able to have a better outcome. Figure 4B shows the performance of the two systems when the critic accuracy was 80%. The top subplot shows when there was no confidence measure and the actor updated every time (S1). The bottom subplot shows the actor updating only when the critic was correct (S2). The critic provided a similar output at the beginning. For the first system, the system started with appropriate random weights and continued to do well with correct critic feedback at the beginning. However, an erroneuous critic feedback at trial 3 caused the system to perform wrong in the next trial. In contrast, the second system started with random weights which caused the first trial to be wrong but the system received good feedback and was able to perform correctly in the subsequent trials. In the first 5 trials, the first system performed better than the second. However, since the second system actor weights were only updated when the critic feedback was good, it took longer for the second system to learn the ideal mapping.

### Neural perturbations—additional noise in data

**Figure 5:**
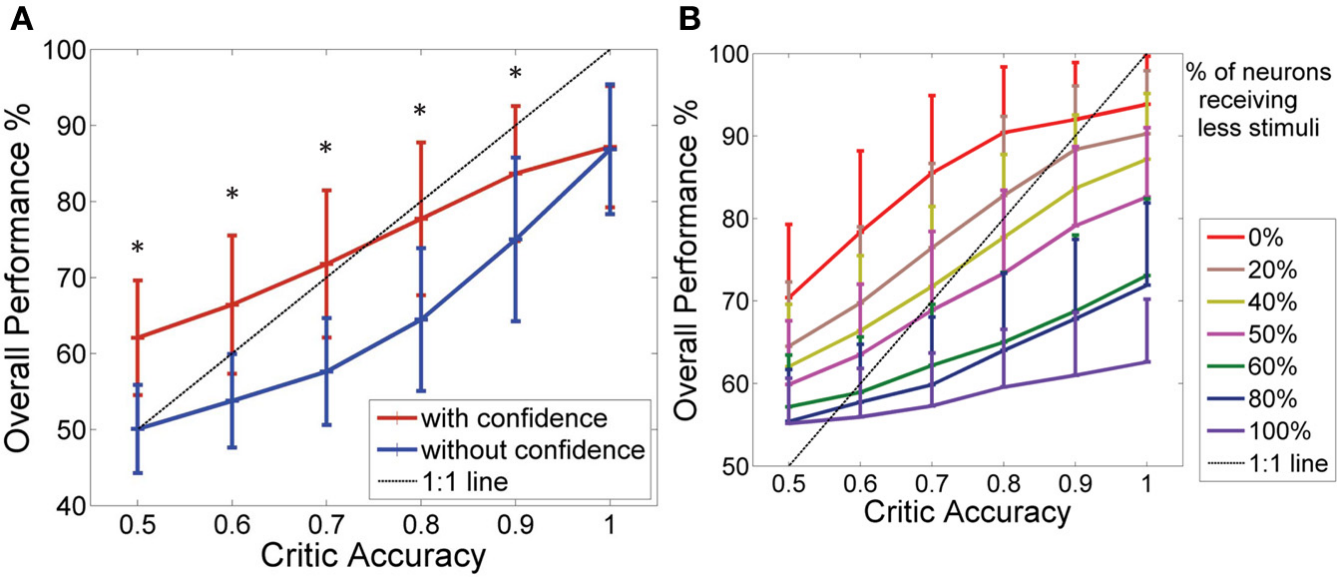
**Effect of noise on the overall performance. (A)** Performance of the BMI Vs the critic accuracy with 40% of the neurons receiving a less stimuli than the standard (mean ± standard deviation. One thousand simulations. One hundred trials per simulation). Red: New update rule with confidence. Blue: Previous method with no confidence. Black: 1:1 relationship. Critic accuracy was varied from 50 to 100% with 100% being the best. ^*^Shows the values which showed statistical significant difference (alpha 0.001). The overall performance of the blue curve is limited by the accuracy of the critic but the overall performance of the red curve is able to go beyond the critic accuracy. Hence, decoupling the performance from the critic accuracy. **(B)** How the overall performance changes with the critic accuracy (1000 simulations). Each curve gives a different noise level of the data set. Percentages indicate the percentage of neurons that were given a less stimuli.

Figure 5A shows how the system with the critic confidence level still performed better than the system which updates the actor weights every time even with the additional noise. At lower critic accuracies, the system which updated at every trial performed at chance level (50% performance), while the system with the critic confidence performed better (at critic accuracies 80% and below the difference in the performance was approximately 10%). However, as the critic accuracy increased (beyond 70%), the system accuracy did not increase as expected in both curves (i.e., both systems stayed below the 1:1 curve). This was due to the limitations in the input data as the data to the decoder was noisy and the states were not as clearly separable. As noted in the previous section, the performance of the two systems showed significant difference for all critic accuracy levels from 50 to 90% (Student's paired *t*-Test, with a two-tailed distribution, alpha 0.001—shown with * in the figure). In Figure 5A, the probability component used to generate *I* was 40%, which was most similar to the NHP data shown in the next section. Figure 5B shows how different noise levels affected the overall performance as the critic accuracy increased. Each colored trace is a different noise level as shown in the legend. With low noise levels, the system was still able to perform amidst the critic inaccuracies. However, as the noise level increased, the system performed at chance (50%) at low critic accuracy levels and performed marginally above chance even at higher critic accuracy levels.

### Simulations using NHP data

**Figure 6:**
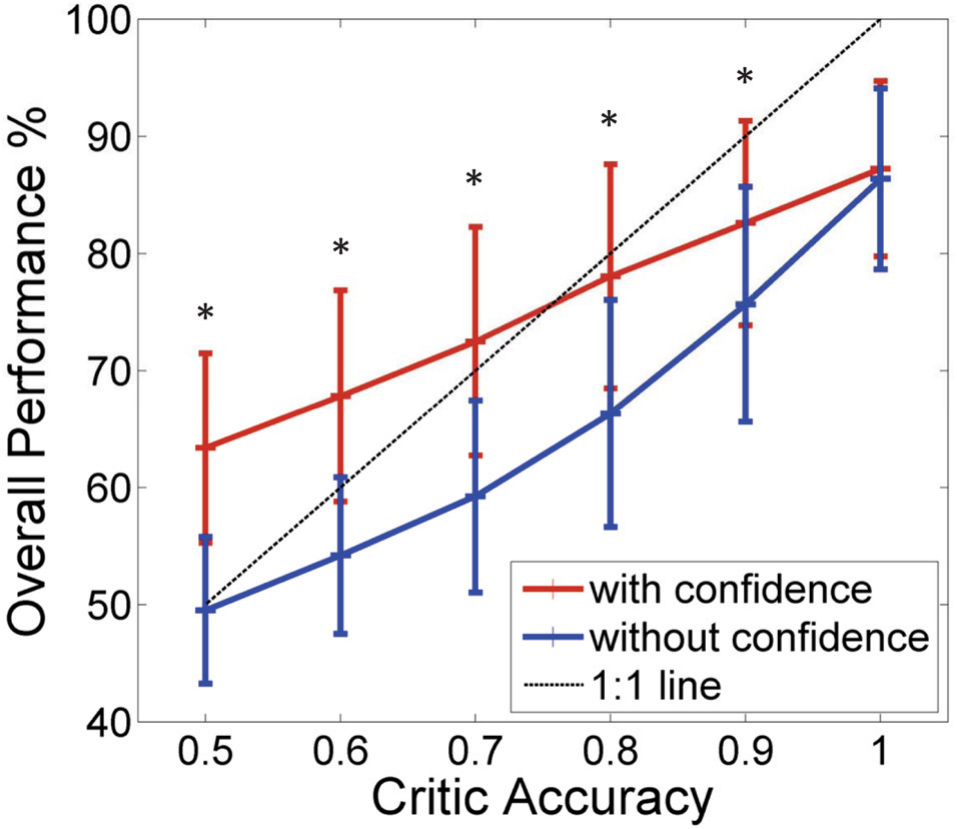
**Results of the simulations where the monkey controls the robot arm**. Performance of the BMI Vs the critic accuracy with and without confidence inbuilt for data collected from monkey DU. (mean ± standard deviation. One thousand simulations). Red: New update rule with confidence. Blue: Previous method with no confidence. Black: 1:1 relationship. Critic accuracy was varied from 50 to 100% with 100% being the best. ^*^Shows the values which showed statistical significant difference (alpha 0.001). At lower critic accuracies, the new update with confidence performs much higher than the one without the confidence measure. As the critic accuracy increase, the plot with the confidence measure is able to outperform the curve without the confidence measure. However, the difference in the performance becomes smaller as the critic accuracy increases suggesting as before that the critic is no longer the limitation, but the nature of the input data itself.

These results are shown in Figure 6 where the blue trace shows the performance of the actor updating every time and the red trace shows the actor updating only when the critic is confident. Similar to the results of the synthetic data, we can see an improvement (from 50 to 63% at critic accuracy of 50% and from 77 to 83% at critic accuracy of 90%) in the overall performance by adding the confidence measure in the update equation. This is more apparent in lower critic accuracies (At alpha = 0.001 critic accuracies 50–90% showed significant difference—shown with ∗ in the figure). At higher critic accuracies, the system which only updates when the critic is confident is still able to do better but the difference in the percentages was smaller. At lower critic accuracies (80% and below) the difference in performance is approximately 13% and at 90% critic accuracy the difference in performance is approximately 7%. Ninety percent critic accuracy means that 9 out of 10 feedback given by the critic is correct. When the critic feedback was always correct, the two systems converged to approximately the same performance value.

## Discussion

In this paper, we demonstrated that adding a confidence level in the feedback to a RL-based decoder can be used to deal with uncertainty in the critic feedback to improve the decoder performance. The introduction of a confidence component in the HRL weight update equation provided guidance on when to update the actor so that the decoder only updated when the feedback was correct with a high confidence. This is important as we seek to utilize biological signals for the critic in order to build autonomous BMIs for use in diverse ADL environments. Preliminary work suggested that the accuracy of extracting this reward signal in animal subjects was less than 90% (Prins et al., 2013) thus indicating that some form of confidence metric will ultimately be needed for real BMI use. In this work, the effects of the critic confidence were tested and the results indicated that the system with the confidence level incorporated outperformed the system without the confidence level at all critic accuracies. This was the case for all 3 different data sets we examined: artificial neural data generated by the Izhikevich method (Izhikevich, 2003), neural data with additional noise, and for data recorded from the MI of a NHP. The system was particularly more effective at lower critic accuracies (<80%). For NHP data the system with the confidence built in performed approximately 13% better than the system without the confidence measure at critic accuracy levels of 50, 60, and 70%. At critic accuracy of 80 and 90%, the system with the confidence performed 12 and 7%, respectively, better than the system without the confidence. For synthetic data with no additional noise, the system with the confidence performed approximately 20% better than the system without the confidence at lower critic accuracies (50, 60, and 70%). At 80% critic accuracy, the difference in performance was 15% and at 90% critic accuracy, this value was 5%. When the critic accuracy was low, updating only when the confidence was high resulted in the actor receiving fewer erroneous feedback, thus causing the system to perform better over time. At higher critic accuracies, since the actor gets correct feedback most of the time, the difference between the two systems, though still noteworthy was small. Both systems converged to the same value when the critic is 100% accurate. As discussed previously, the neural data proposed for the critic input yielded less than perfect accuracies which made it necessary to find an alternate way to deal with the actor update rule.

### Noisy neural data

Noisy neural signals as well as complex neural representation of reward make it a challenging task to classify rewarding vs. non rewarding information with a high accuracy (Schultz et al., 1997; O'doherty, 2004; Knutson et al., 2005). Building a confidence in to the critic feedback improved the performance of the system when the data was contaminated with noise and when the multiple neural representations caused difficultly in extracting a single feedback signal required by the actor-critic decoder. We tested how overlapping classes in the motor data can influence the ability of the decoder to predict the correct action; more the classes overlap, lesser the accuracy in decoding. To add noise to the data, we used a Gaussian distribution in the stimulating current, which resulted in reducing the stimulating current of a certain percentage of neurons in the ensembles that were already tuned. Here, we also showed that with limited noise in the motor data, the system was able to maintain performance. When the motor neural data was noisy, the limiting factor became how well the motor neural data represented the task.

### Overcoming inherent issues with RL—time for convergence

Due to the inherent nature of RL that learns through interaction, the time taken to reach an optimal condition in the weights can longer than for supervised decoders (Beggs, 2005). The agent needs to “explore” its environment in order to have a better understanding of how each action changes the state of the environment. Once the agent has learned enough about the environment, it will “exploit” the situation or carry out the optimal action. In RL, there is always a dilemma between exploration and exploitation. Before the agent knows the optimal action and exploit it, the agent has to make several sub-optimal actions in order to explore the environment. The more exploration that takes place, the better understanding it will have of its environment, but the longer it will take to reach an optimal solution. In the case of BMIs, the agent does not have many trials to explore as each trial comes at a cost. Due to this, the experience of an agent in the BMI setting is very limited. In previous studies, we have used real time “epoching” of the data to speed the initial adaptation from the purely random initialization weights to functionally useful ones as a method of increasing experience with limited data. Another method for overcoming RL limitations is to use a memory of past trials. Here, we used a memory size of 1 trial. For more complicated tasks, a memory size of 70 trials has been found out to give the optimum results (Mahmoudi et al., 2013; Pohlmeyer et al., 2014).

### Extracting optimal reward signal for biological critic feedback

There are several regions of the brain that can be used to extract a reward signal for the critic, which include the striatum (Phillips, 1984; Wise and Bozarth, 1984; Wise and Rompré, 1989; Schultz et al., 1992; Tanaka et al., 2004), cingulate (Shima and Tanji, 1998; Bush et al., 2002; Shidara and Richmond, 2002), and orbitofrontal cortices (Rolls, 2000; Schultz et al., 2000; Tremblay and Schultz, 2000). Whichever region is selected, the critic will need to decode the reward as well as the confidence it has in its decision. One possible method of decoding the confidence is using the distance to the boundary of a decision surface: the closer a data point is to the decision boundary, the less confidence it has in its decision and further away the data point is, the more confidence it has in its decision. This method assumes that the misclassifications are due to overlapping classes and not due to mislabeled trials. This concept will be further developed in future work.

In this paper, we developed a new formulation for an actor-critic BMI decoder in order to be able to use biological feedback signals. Since RL does not need an explicit training signal to train the decoder, it can be used to develop next-generation BMIs that self-calibrate in scenarios where the user is paralyzed and cannot generate a kinematic reference or training signal. The actor-critic RL paradigm also gives us the flexibility to develop a fully autonomous BMI provided the critic can be driven by a biological source and thus reduce set up times and the need for calibrations.

### Conflict of interest statement

The authors declare that the research was conducted in the absence of any commercial or financial relationships that could be construed as a potential conflict of interest.

## References

[B1] Andersen R. A. Musallam S. Pesaran B. (2004). Selecting the signals for a brain–machine interface. Curr. Opin. Neurobiol. 14, 720–726 10.1016/j.conb.2004.10.005 15582374

[B2] Antoni J. Randall R. (2004). Unsupervised noise cancellation for vibration signals: part I—Evaluation of adaptive algorithms. Mech. Syst. Signal Process. 18, 89–101 10.1016/S0888-3270(03)00012-8

[B3] Apicella P. Ljungberg T. Scarnati E. Schultz W. (1991). Responses to reward in monkey dorsal and ventral striatum. Exp. Brain Res. 85, 491–500 10.1007/BF00231732 1915708

[B4] Bai O. Lin P. Huang D. Fei D.-Y. Floeter M. K. (2010). Towards a user-friendly brain–computer interface: initial tests in ALS and PLS patients. Clin. Neurophysiol. 121, 1293–1303 10.1016/j.clinph.2010.02.157 20347612 PMC2895010

[B5] Barto A. Anandan P. (1985). Pattern-recognizing stochastic learning automata. IEEE Trans. Syst. Man Cybern. SMC-15, 360–375 10.1109/TSMC.1985.6313371

[B6] Bashashati A. Fatourechi M. Ward R. K. Birch G. E. (2007). A survey of signal processing algorithms in brain–computer interfaces based on electrical brain signals. J. Neural Eng. 4, R32 10.1088/1741-2560/4/2/R03 17409474

[B7] Beggs A. W. (2005). On the convergence of reinforcement learning. J. Econ. Theory 122, 1–36 10.1016/j.jet.2004.03.008

[B8] Birbaumer N. Cohen L. G. (2007). Brain–computer interfaces: communication and restoration of movement in paralysis. J. Physiol. 579, 621–636 10.1113/jphysiol.2006.125633 17234696 PMC2151357

[B9] Bosman R. Van Leeuwen W. Wemmenhove B. (2004). Combining Hebbian and reinforcement learning in a minibrain model. Neural Netw. 17, 29–36 10.1016/j.neunet.2003.07.007 14690704

[B10] Bush G. Vogt B. A. Holmes J. Dale A. M. Greve D. Jenike M. A. (2002). Dorsal anterior cingulate cortex: a role in reward-based decision making. Proc. Natl. Acad. Sci. U.S.A. 99, 523–528 10.1073/pnas.012470999 11756669 PMC117593

[B11] Chestek C. A. Gilja V. Nuyujukian P. Foster J. D. Fan J. M. Kaufman M. T. (2011). Long-term stability of neural prosthetic control signals from silicon cortical arrays in rhesus macaque motor cortex. J. Neural Eng. 8:045005 10.1088/1741-2560/8/4/045005 21775782 PMC3644617

[B12] Collinger J. L. Wodlinger B. Downey J. E. Wang W. Tyler-Kabara E. C. Weber D. J. (2013). High-performance neuroprosthetic control by an individual with tetraplegia. Lancet. 381, 557–564 10.1016/S0140-6736(12)61816-9 23253623 PMC3641862

[B13] Daly J. J. Wolpaw J. R. (2008). Brain–computer interfaces in neurological rehabilitation. Lancet Neurol. 7, 1032–1043 10.1016/S1474-4422(08)70223-0 18835541

[B14] Di Pino G. Porcaro C. Tombini M. Assenza G. Pellegrino G. Tecchio F. (2012). A neurally-interfaced hand prosthesis tuned inter-hemispheric communication. Restor. Neurol. Neurosci. 30, 407–418 10.3233/RNN-2012-120224 22751356

[B15] Doya K. (2000). Complementary roles of basal ganglia and cerebellum in learning and motor control. Curr. Opin. Neurobiol. 10, 732–739 10.1016/S0959-4388(00)00153-7 11240282

[B16] Elbert T. Rockstroh B. (2004). Reorganization of human cerebral cortex: the range of changes following use and injury. Neuroscientist 10, 129–141 10.1177/1073858403262111 15070487

[B17] Ethier C. Oby E. Bauman M. Miller L. (2012). Restoration of grasp following paralysis through brain-controlled stimulation of muscles. Nature 485, 368–371 10.1038/nature10987 22522928 PMC3358575

[B18] Fisher J. Principe J. (1996). Unsupervised learning for nonlinear synthetic discriminant functions. Proc. SPIE 2752, 2–13 10.1117/12.235636

[B19] Flint R. D. Wright Z. A. Scheid M. R. Slutzky M. W. (2013). Long term, stable brain machine interface performance using local field potentials and multiunit spikes. J. Neural Eng. 10, 056005 10.1088/1741-2560/10/5/056005 23918061 PMC4023629

[B20] Gilja V. Nuyujukian P. Chestek C. A. Cunningham J. P. Byron M. Y. Fan J. M. (2012). A high-performance neural prosthesis enabled by control algorithm design. Nat. Neurosci. 15, 1752–1757 10.1038/nn.3265 23160043 PMC3638087

[B21] Gullapalli V. (1991). Associative reinforcement learning of real-valued functions, in 1991 IEEE International Conference on Systems, Man, and Cybernetics. Decision Aiding for Complex Systems, (Charlottesville, VA), 1453–1458 10.1109/ICSMC.1991.169893

[B22] Gürel T. Mehring C. (2012). Unsupervised adaptation of brain-machine interface decoders. Front. Neurosci. 6:164 10.3389/fnins.2012.00164 23162425 PMC3499737

[B23] Hochberg L. R. Bacher D. Jarosiewicz B. Masse N. Y. Simeral J. D. Vogel J. (2012). Reach and grasp by people with tetraplegia using a neurally controlled robotic arm. Nature 485, 372–375 10.1038/nature11076 22596161 PMC3640850

[B24] Hochberg L. R. Serruya M. D. Friehs G. M. Mukand J. A. Saleh M. Caplan A. H. (2006). Neuronal ensemble control of prosthetic devices by a human with tetraplegia. Nature 442, 164–171 10.1038/nature04970 16838014

[B25] Hollerman J. R. Tremblay L. Schultz W. (1998). Influence of reward expectation on behavior-related neuronal activity in primate striatum. J. Neurophysiol. 80, 947–963 9705481 10.1152/jn.1998.80.2.947

[B26] Houk J. C. Wise S. P. (1995). Distributed modular architectures linking basal ganglia, cerebellum, and cerebral cortex: their role in planning and controlling action. Cereb. Cortex 5, 95–110 10.1093/cercor/5.2.95 7620294

[B27] Izhikevich E. M. (2003). Simple model of spiking neurons. IEEE Trans. Neural Netw. 14, 1569–1572 10.1109/TNN.2003.820440 18244602

[B28] Kaelbling L. P. (1994). Associative reinforcement learning: a generate and test algorithm. Mach. Learn. 15, 299–319 10.1007/BF00993348

[B29] Kelley A. E. (2004). Ventral striatal control of appetitive motivation: role in ingestive behavior and reward-related learning. Neurosci. Biobehav. Rev. 27, 765–776 10.1016/j.neubiorev.2003.11.015 15019426

[B30] Kim S. Sanchez J. Rao Y. Erdogmus D. Carmena J. Lebedev M. (2006). A comparison of optimal MIMO linear and nonlinear models for brain–machine interfaces. J. Neural Eng. 3, 145 10.1088/1741-2560/3/2/009 16705271

[B31] Knutson B. Taylor J. Kaufman M. Peterson R. Glover G. (2005). Distributed neural representation of expected value. J. Neurosci. 25, 4806–4812 10.1523/JNEUROSCI.0642-05.2005 15888656 PMC6724773

[B32] Li Z. O'doherty J. E. Lebedev M. A. Nicolelis M. A. (2011). Adaptive decoding for brain-machine interfaces through Bayesian parameter updates. Neural Comput. 23, 3162–3204 10.1162/NECO_a_00207 21919788 PMC3335277

[B33] Mahmoudi B. Pohlmeyer E. A. Prins N. W. Geng S. Sanchez J. C. (2013). Towards autonomous neuroprosthetic control using Hebbian reinforcement learning. J. Neural Eng. 10, 066005 10.1088/1741-2560/10/6/066005 24100047

[B34] Mahmoudi B. Sanchez J. C. (2011). A symbiotic brain-machine interface through value-based decision making. PLoS ONE 6:e14760 10.1371/journal.pone.0014760 21423797 PMC3056711

[B35] Maynard E. M. Nordhausen C. T. Normann R. A. (1997). The Utah intracortical electrode array: a recording structure for potential brain-computer interfaces. Electroencephalogr. Clin. Neurophysiol. 102, 228–239 10.1016/S0013-4694(96)95176-0 9129578

[B36] Mehring C. Rickert J. Vaadia E. De Oliveira S. C. Aertsen A. Rotter S. (2003). Inference of hand movements from local field potentials in monkey motor cortex. Nat. Neurosci. 6, 1253–1254 10.1038/nn1158 14634657

[B37] Miller L. E. Weber D. J. (2011). Guest editorial brain training: cortical plasticity and afferent feedback in brain-machine interface systems. IEEE Trans. Neural Syst. Rehabil. Eng. 19, 465–467 10.1109/TNSRE.2011.2168989 21947530

[B38] Moritz C. T. Perlmutter S. I. Fetz E. E. (2008). Direct control of paralysed muscles by cortical neurons. Nature 456, 639–642 10.1038/nature07418 18923392 PMC3159518

[B39] Nicola S. M. (2007). The nucleus accumbens as part of a basal ganglia action selection circuit. Psychopharmacology 191, 521–550 10.1007/s00213-006-0510-4 16983543

[B40] O'doherty J. P. (2004). Reward representations and reward-related learning in the human brain: insights from neuroimaging. Curr. Opin. Neurobiol. 14, 769–776 10.1016/j.conb.2004.10.016 15582382

[B41] Patil P. G. Turner D. A. (2008). The development of brain-machine interface neuroprosthetic devices. Neurotherapeutics 5, 137–146 10.1016/j.nurt.2007.11.002 18164493 PMC5084136

[B42] Pennartz C. (1997). Reinforcement learning by Hebbian synapses with adaptive thresholds. Neuroscience 81, 303–319 10.1016/S0306-4522(97)00118-8 9300423

[B43] Pennartz C. Groenewegen H. J. Da Silva F. (1994). The nucleus accumbens as a complex of functionally distinct neuronal ensembles: an integration of behavioural, electrophysiological and anatomical data. Prog. Neurobiol. 42, 719–761 10.1016/0301-0082(94)90025-6 7938546

[B44] Pfurtscheller G. Neuper C. (2001). Motor imagery and direct brain-computer communication. Proc. IEEE 89, 1123–1134 10.1109/5.939829

[B45] Phillips A. G. (1984). Brain reward circuitry: a case for separate systems. Brain Res. Bull. 12, 195–201 10.1016/0361-9230(84)90189-8 6609750

[B46] Pohlmeyer E. A. Mahmoudi B. Geng S. J. Prins N. W. Sanchez J. C. (2014). Using Reinforcement learning to provide stable brain-machine interface control despite neural input reorganization. PLoS ONE 9:e87253 10.1371/journal.pone.0087253 24498055 PMC3907465

[B47] Pohlmeyer E. A. Oby E. R. Perreault E. J. Solla S. A. Kilgore K. L. Kirsch R. F. (2009). Toward the restoration of hand use to a paralyzed monkey: brain-controlled functional electrical stimulation of forearm muscles. PLoS ONE 4:e5924 10.1371/journal.pone.0005924 19526055 PMC2691481

[B48] Prasad A. Xue Q.-S. Sankar V. Nishida T. Shaw G. Streit W. J. (2012). Comprehensive characterization and failure modes of tungsten microwire arrays in chronic neural implants. J. Neural Eng. 9, 056015 10.1088/1741-2560/9/5/056015 23010756

[B49] Prins N. W. Geng S. Pohlmeyer E. A. Mahmoudi B. Sanchez J. C. (2013). Feature extraction and unsupervised classification of neural population reward signals for reinforcement based BMI, in 2013 35th Annual International Conference of the IEEE on Engineering in Medicine and Biology Society (EMBC), (Osaka), 5250–5253 10.1109/EMBC.2013.6610733 24110920

[B50] Rao R. P. (2010). Decision making under uncertainty: a neural model based on partially observable Markov decision processes. Front. Comput. Neurosci. 4:146 10.3389/fncom.2010.00146 21152255 PMC2998859

[B51] Rolls E. T. (2000). The orbitofrontal cortex and reward. Cereb. Cortex 10, 284–294 10.1093/cercor/10.3.284 10731223

[B52] Sanchez J. C. Carmena J. M. Lebedev M. A. Nicolelis M. A. Harris J. G. Principe J. C. (2004). Ascertaining the importance of neurons to develop better brain-machine interfaces. IEEE Trans. Biomed. Eng. 51, 943–953 10.1109/TBME.2004.827061 15188862

[B53] Sanes J. N. Donoghue J. P. (2000). Plasticity and primary motor cortex. Annu. Rev. Neurosci. 23, 393–415 10.1146/annurev.neuro.23.1.393 10845069

[B54] Santhanam G. Ryu S. I. Byron M. Y. Afshar A. Shenoy K. V. (2006). A high-performance brain–computer interface. Nature 442, 195–198 10.1038/nature04968 16838020

[B55] Schalk G. Kubanek J. Miller K. Anderson N. Leuthardt E. Ojemann J. (2007). Decoding two-dimensional movement trajectories using electrocorticographic signals in humans. J. Neural Eng. 4, 264 10.1088/1741-2560/4/3/012 17873429

[B56] Schultz W. Apicella P. Scarnati E. Ljungberg T. (1992). Neuronal activity in monkey ventral striatum related to the expectation of reward. J. Neurosci. 12, 4595–4610 1464759 10.1523/JNEUROSCI.12-12-04595.1992 PMC6575755

[B57] Schultz W. Dayan P. Montague P. R. (1997). A neural substrate of prediction and reward. Science 275, 1593–1599 10.1126/science.275.5306.1593 9054347

[B58] Schultz W. Tremblay L. Hollerman J. R. (2000). Reward processing in primate orbitofrontal cortex and basal ganglia. Cereb. Cortex 10, 272–283 10.1093/cercor/10.3.272 10731222

[B59] Schwartz A. B. Cui X. T. Weber D. J. Moran D. W. (2006). Brain-controlled interfaces: movement restoration with neural prosthetics. Neuron 52, 205–220 10.1016/j.neuron.2006.09.019 17015237

[B60] Shenoy P. Krauledat M. Blankertz B. Rao R. P. Müller K.-R. (2006). Towards adaptive classification for BCI. J. Neural Eng. 3, R13 10.1088/1741-2560/3/1/R02 16510936

[B61] Shenoy P. Rao R. P. (2005). Dynamic bayesian networks for brain-computer interfaces, in Advances in Neural Information Processing Systems (Vancouver, BC: MIT Press), 1265–1272

[B62] Shidara M. Richmond B. J. (2002). Anterior cingulate: single neuronal signals related to degree of reward expectancy. Science 296, 1709–1711 10.1126/science.1069504 12040201

[B63] Shima K. Tanji J. (1998). Role for cingulate motor area cells in voluntary movement selection based on reward. Science 282, 1335–1338 10.1126/science.282.5392.1335 9812901

[B64] Shoham S. Paninski L. M. Fellows M. R. Hatsopoulos N. G. Donoghue J. P. Normann R. A. (2005). Statistical encoding model for a primary motor cortical brain-machine interface. IEEE Trans. Biomed. Eng. 52, 1312–1322 10.1109/TBME.2005.847542 16041995

[B65] Simeral J. Kim S. Black M. Donoghue J. Hochberg L. (2011). Neural control of cursor trajectory and click by a human with tetraplegia 1000 days after implant of an intracortical microelectrode array. J. Neural Eng. 8, 025027 10.1088/1741-2560/8/2/025027 21436513 PMC3715131

[B66] Snider R. Bonds A. (1998). Classification of non-stationary neural signals. J. Neurosci. Methods 84, 155–166 10.1016/S0165-0270(98)00110-1 9821647

[B67] Sutton R. S. Barto A. G. (1998). Reinforcement Learning: An Introduction, Cambridge, MA: MIT Press

[B68] Tanaka S. C. Doya K. Okada G. Ueda K. Okamoto Y. Yamawaki S. (2004). Prediction of immediate and future rewards differentially recruits cortico-basal ganglia loops. Nat. Neurosci. 7, 887–893 10.1038/nn1279 15235607

[B69] Tremblay L. Schultz W. (2000). Reward-related neuronal activity during go-nogo task performance in primate orbitofrontal cortex. J. Neurophysiol. 83, 1864–1876 10.1234/12345678 10758098

[B70] Vidaurre C. Kawanabe M. Von Bunau P. Blankertz B. Muller K. (2011). Toward unsupervised adaptation of LDA for brain–computer interfaces. IEEE Trans. Biomed. Eng. 58, 587–597 10.1109/TBME.2010.2093133 21095857

[B71] Wessberg J. Stambaugh C. R. Kralik J. D. Beck P. D. Laubach M. Chapin J. K. (2000). Real-time prediction of hand trajectory by ensembles of cortical neurons in primates. Nature 408, 361–365 10.1038/35042582 11099043

[B72] Wise R. A. Bozarth M. A. (1984). Brain reward circuitry: Four circuit elements “wired” in apparent series. Brain Res. Bull. 12, 203–208 10.1016/0361-9230(84)90190-4 6609751

[B73] Wise R. A. Rompré P.-P. (1989). Brain dopamine and reward. Annu. Rev. Psychol. 40, 191–225 10.1146/annurev.ps.40.020189.001203 2648975

[B74] Wolpaw J. R. McFarland D. J. (2004). Control of a two-dimensional movement signal by a noninvasive brain-computer interface in humans. Proc. Natl. Acad. Sci. U.S.A. 101, 17849–17854 10.1073/pnas.0403504101 15585584 PMC535103

