# Supplementary material for: A confidence metric for using neurobiological feedback in actor-critic reinforcement learning based brain-machine interfaces
Source: Front Neurosci. 2014 May 26;8:111. doi: 10.3389/fnins.2014.00111 (PMC4033619; doi:10.3389/fnins.2014.00111)
Supplement: Supplementary file 1 [file Presentation1.PDF]

Electrode impedance analysis of chronic tungsten microwire neural implants: understanding abiotic vs. biotic contributions

Viswanath Sankar, Erin Patrick, Robert Dieme, Justin C. Sanchez, Abhishek Prasad*, and Toshikazu Nishida

[1]Electrical and Computer Engineering Department, University of Florida, Gainesville, FL, USA
[2]Biomedical Engineering Department, University of Miami, Coral Gables, FL, USA

*** Correspondence:** Abhishek Prasad
Department of Biomedical Engineering, University of Miami, 1251 Memorial Drive, Rm#203, Coral Gables FL 33146. Email: a.prasad@miami.edu. Phone: 305-284-4886

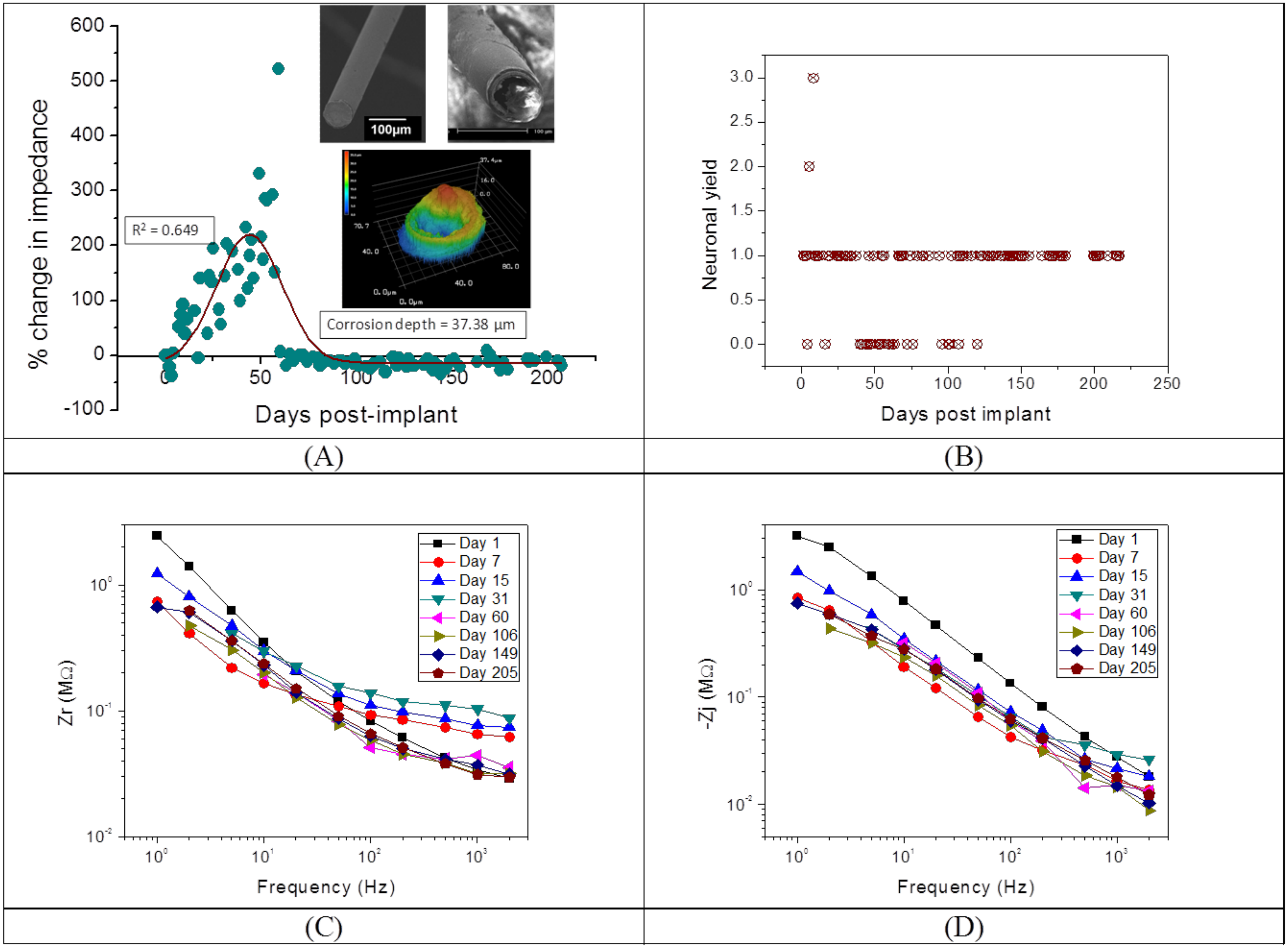

Figure 1: Wire 1 plots. A) Percentage change in in-vivo impedance plotted against the implanted duration and fitted with a Gaussian curve, B) Neuronal yield during the implanted period, C) Real part of the impedance across the measured frequency spectrum, and D) Imaginary part of the impedance across the measured frequency spectrum.

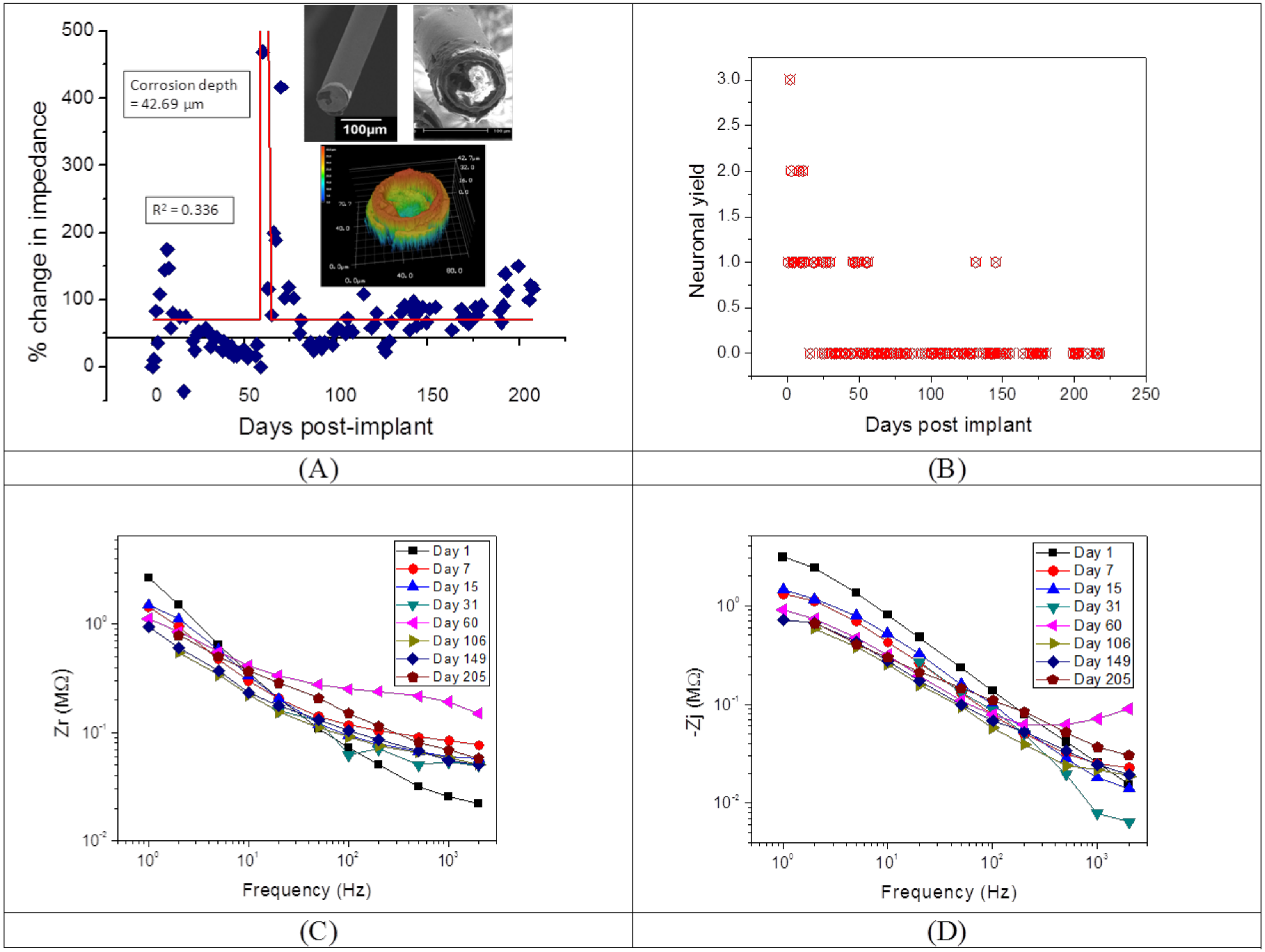

Figure 2: Wire 2 plots. A) Percentage change in in-vivo impedance plotted against the implanted duration and fitted with a Gaussian curve, B) Neuronal yield during the implanted period, C) Real part of the impedance across the measured frequency spectrum, and D) Imaginary part of the impedance across the measured frequency spectrum.

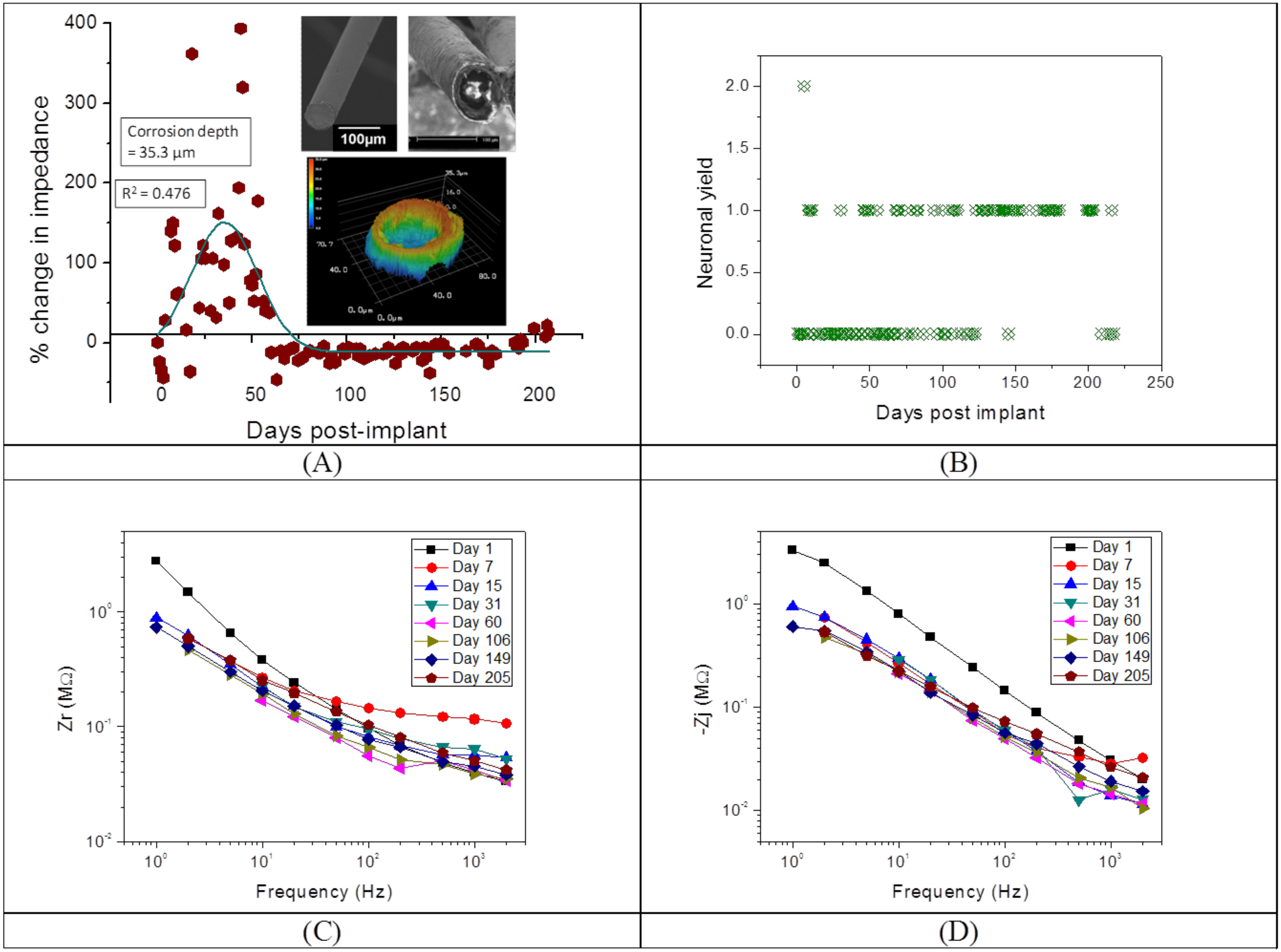

Figure 3: Wire 3 plots. A) Percentage change in in-vivo impedance plotted against the implanted duration and fitted with a Gaussian curve, B) Neuronal yield during the implanted period, C) Real part of the impedance across the measured frequency spectrum, and D) Imaginary part of the impedance across the measured frequency spectrum.

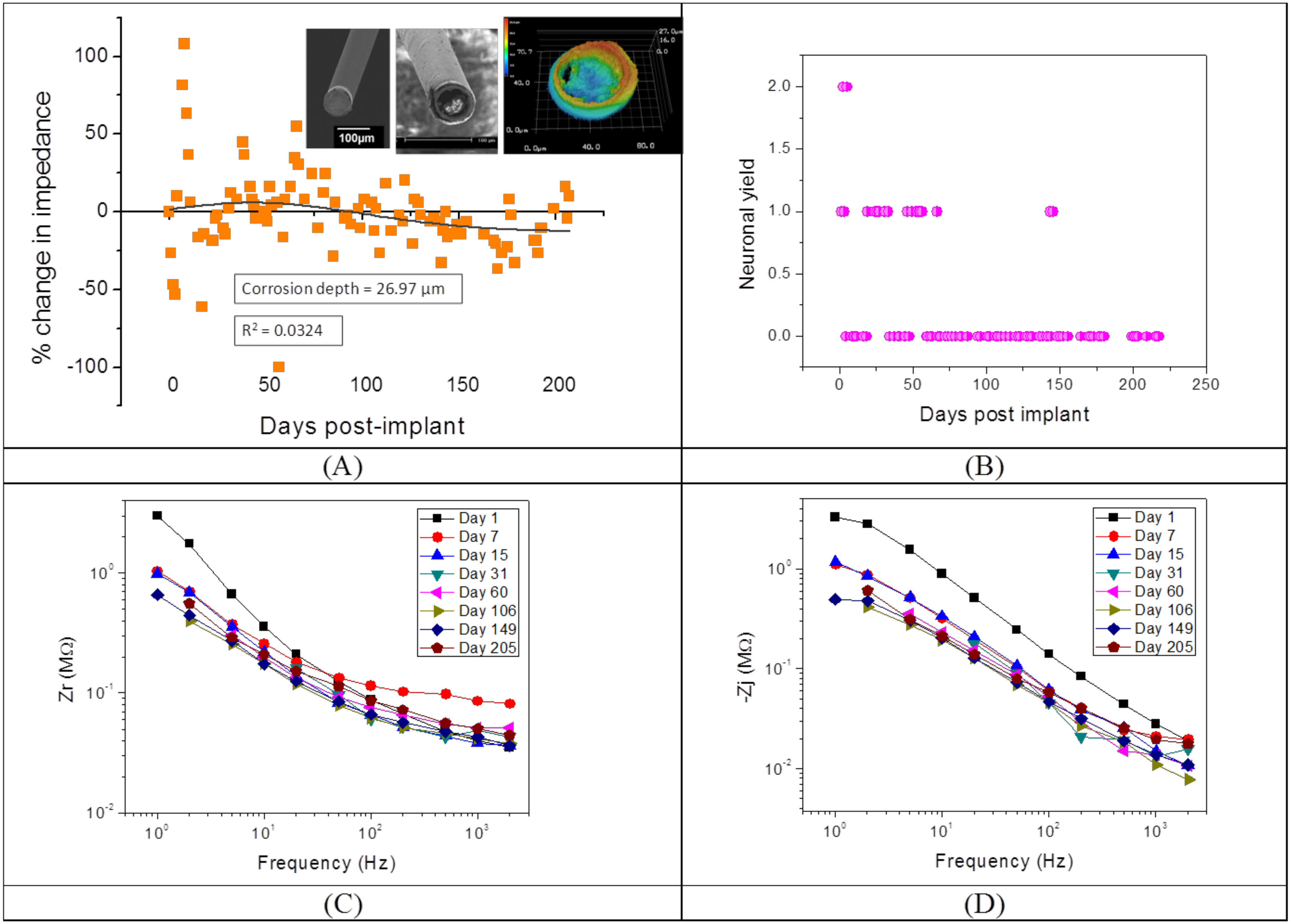

Figure 4: Wire 4 plots. A) Percentage change in in-vivo impedance plotted against the implanted duration and fitted with a Gaussian curve, B) Neuronal yield during the implanted period, C) Real part of the impedance across the measured frequency spectrum, and D) Imaginary part of the impedance across the measured frequency spectrum.

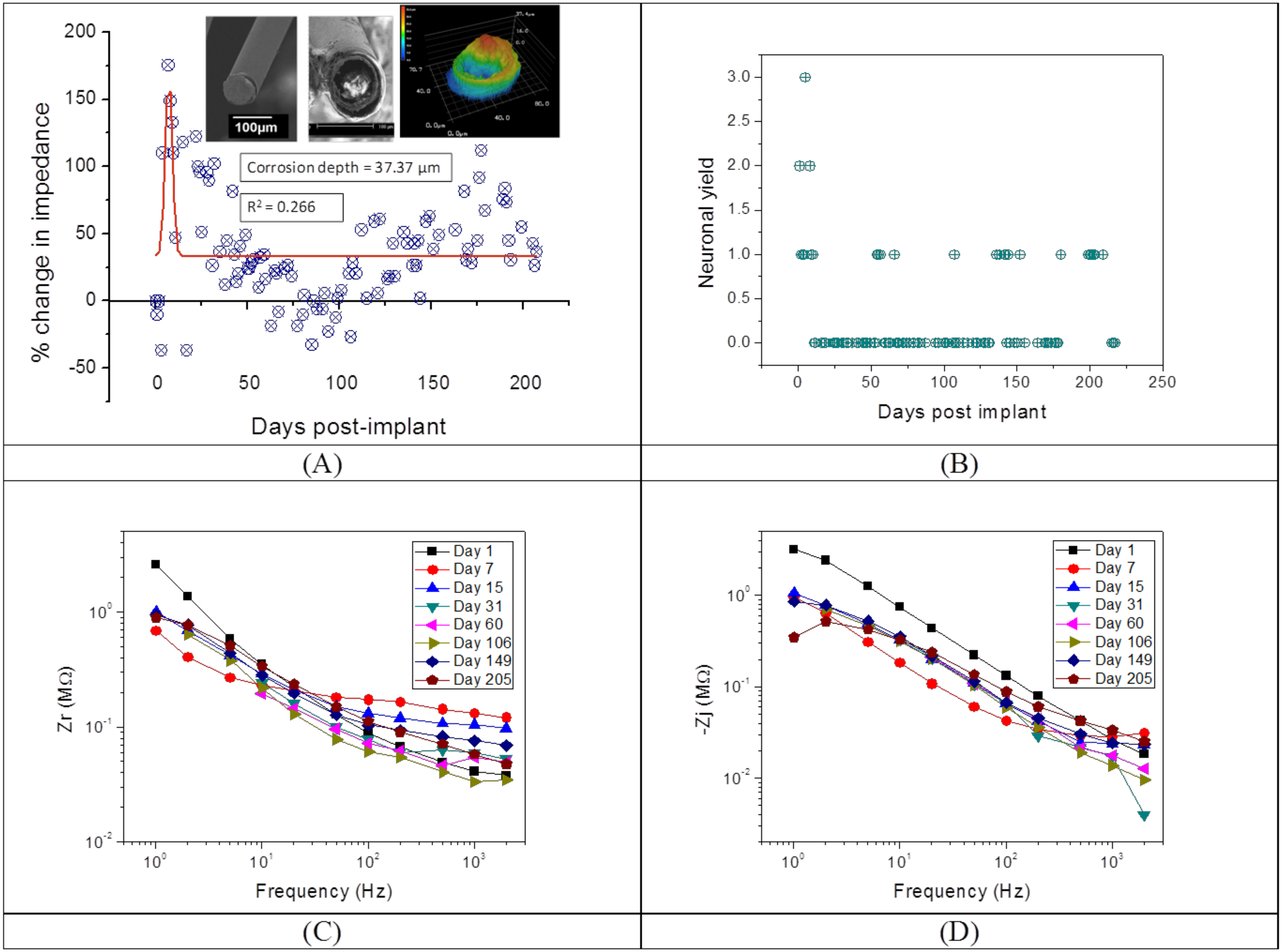

Figure 5: Wire 5 plots. A) Percentage change in in-vivo impedance plotted against the implanted duration and fitted with a Gaussian curve, B) Neuronal yield during the implanted period, C) Real part of the impedance across the measured frequency spectrum, and D) Imaginary part of the impedance across the measured frequency spectrum.

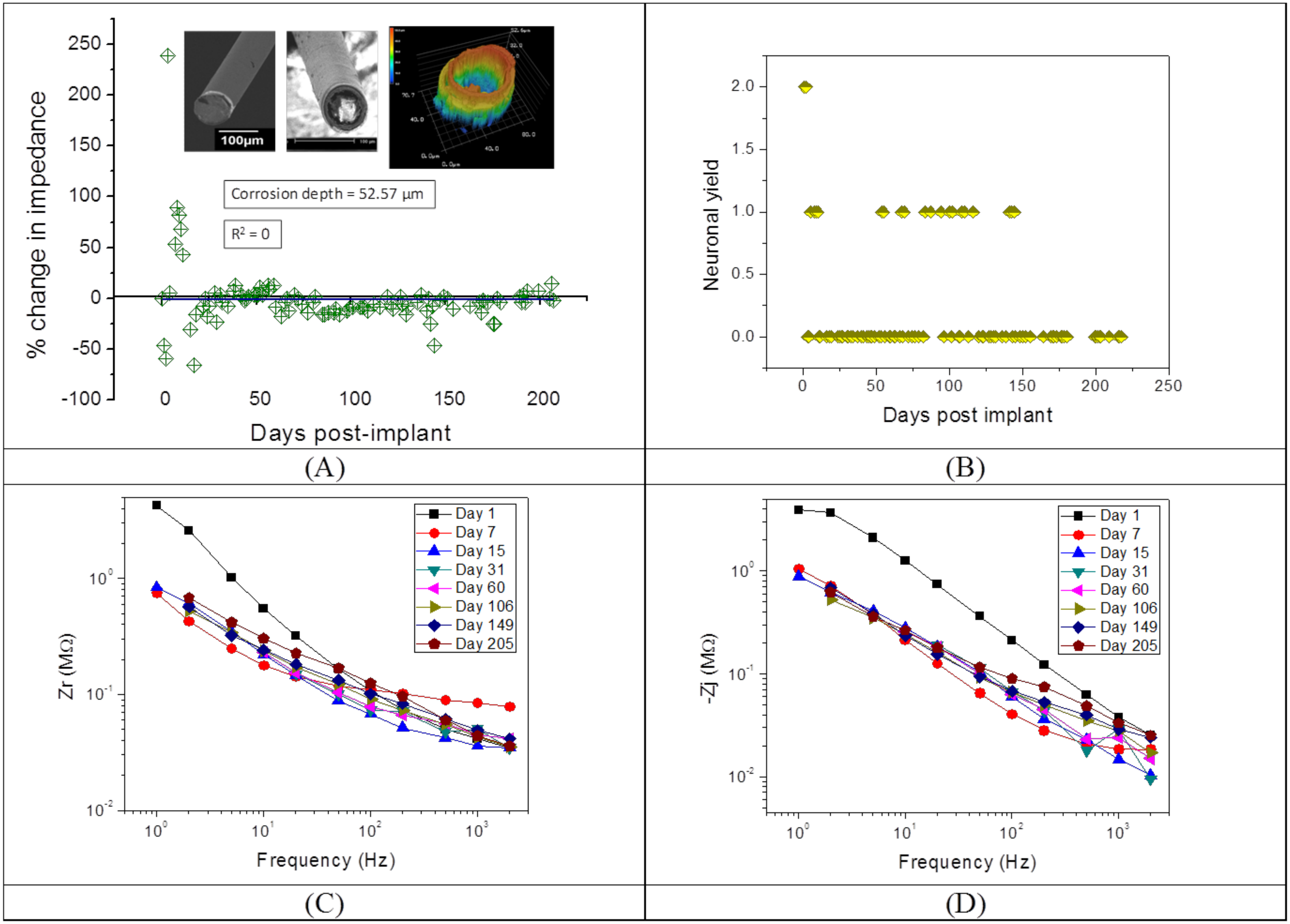

Figure 6: Wire 6 plots. A) Percentage change in in-vivo impedance plotted against the implanted duration and fitted with a Gaussian curve, B) Neuronal yield during the implanted period, C) Real part of the impedance across the measured frequency spectrum, and D) Imaginary part of the impedance across the measured frequency spectrum.

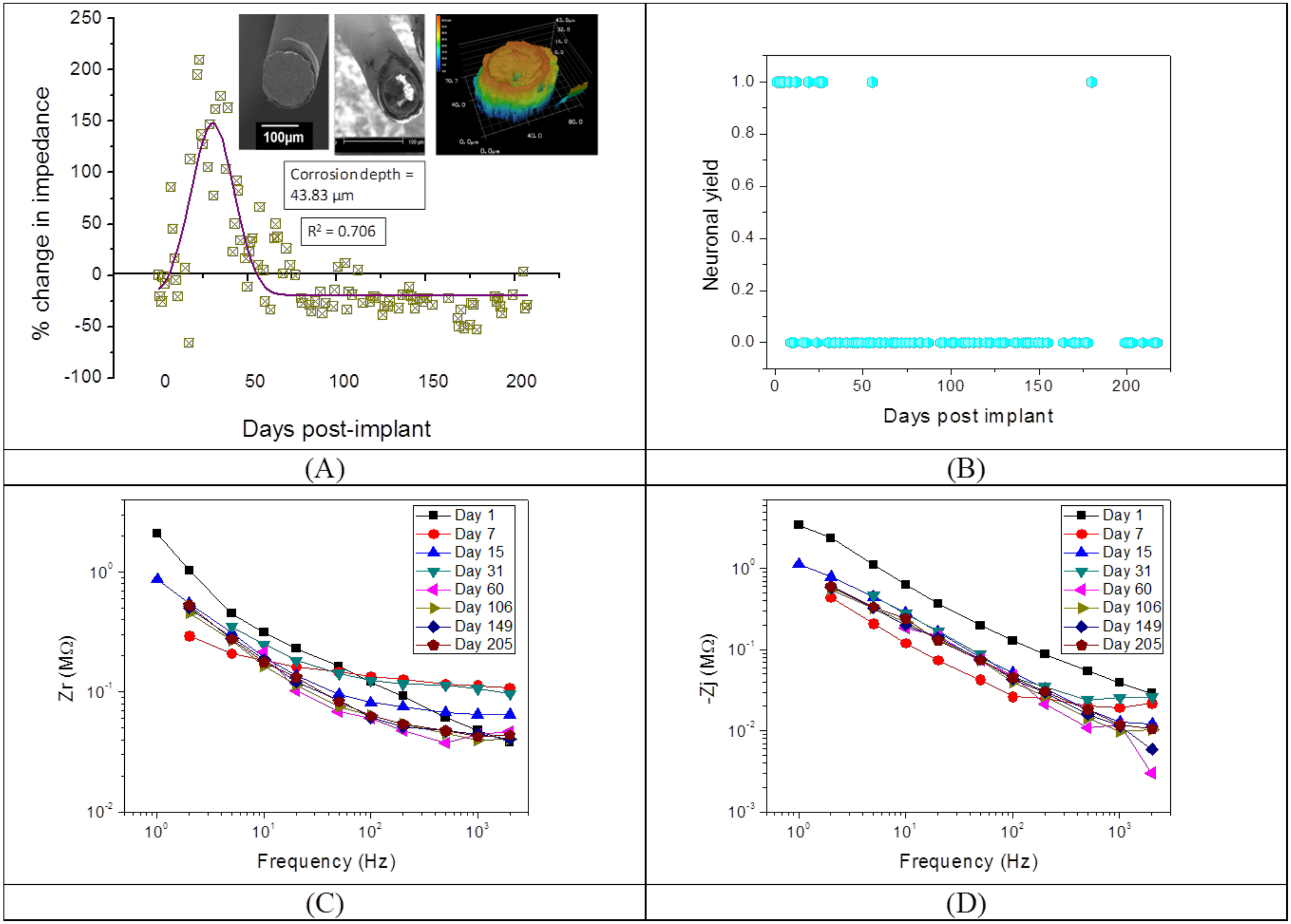

Figure 7: Wire 7 plots. A) Percentage change in in-vivo impedance plotted against the implanted duration and fitted with a Gaussian curve, B) Neuronal yield during the implanted period, C) Real part of the impedance across the measured frequency spectrum, and D) Imaginary part of the impedance across the measured frequency spectrum.

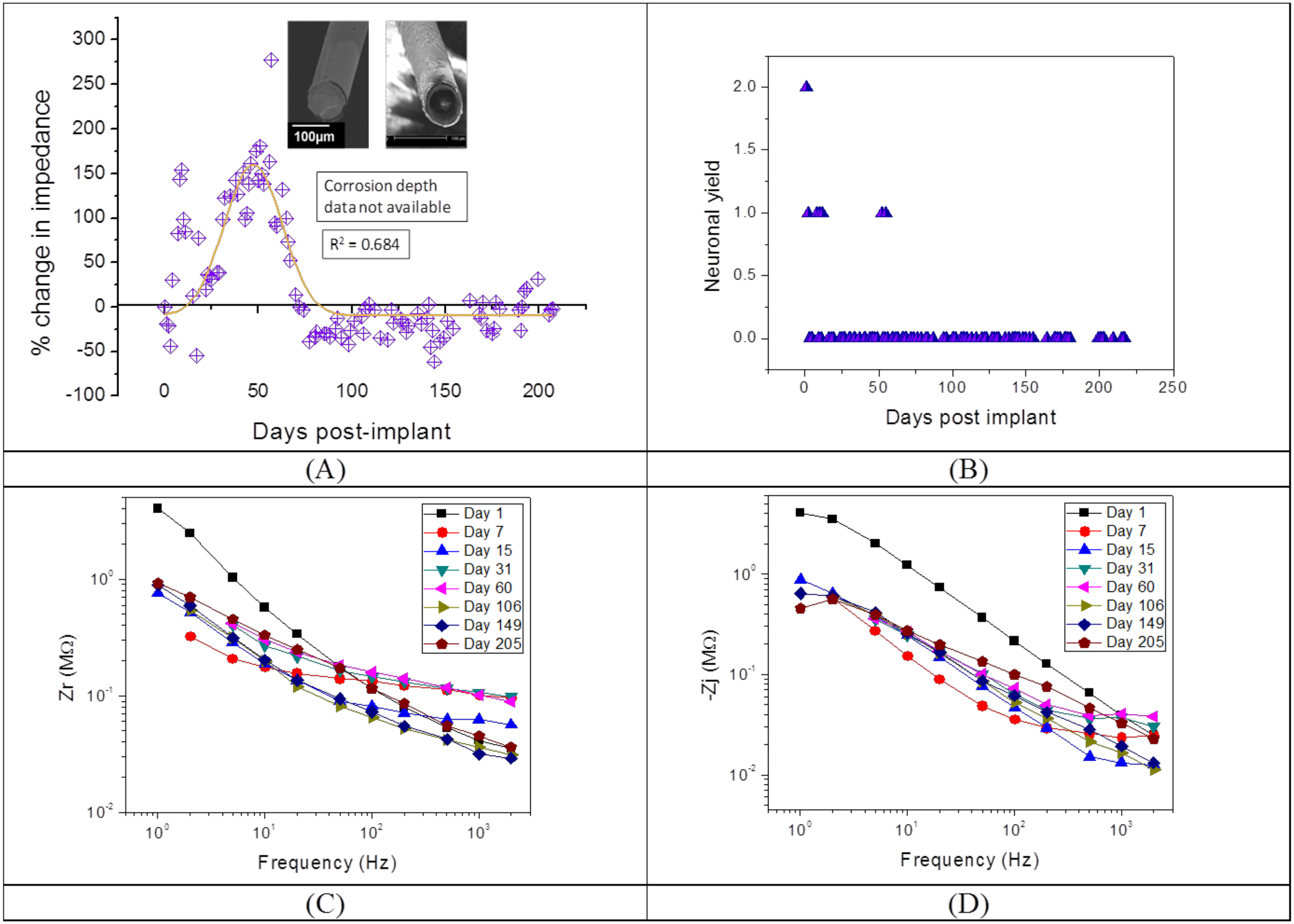

Figure 8: Wire 8 plots. A) Percentage change in in-vivo impedance plotted against the implanted duration and fitted with a Gaussian curve, B) Neuronal yield during the implanted period, C) Real part of the impedance across the measured frequency spectrum, and D) Imaginary part of the impedance across the measured frequency spectrum.

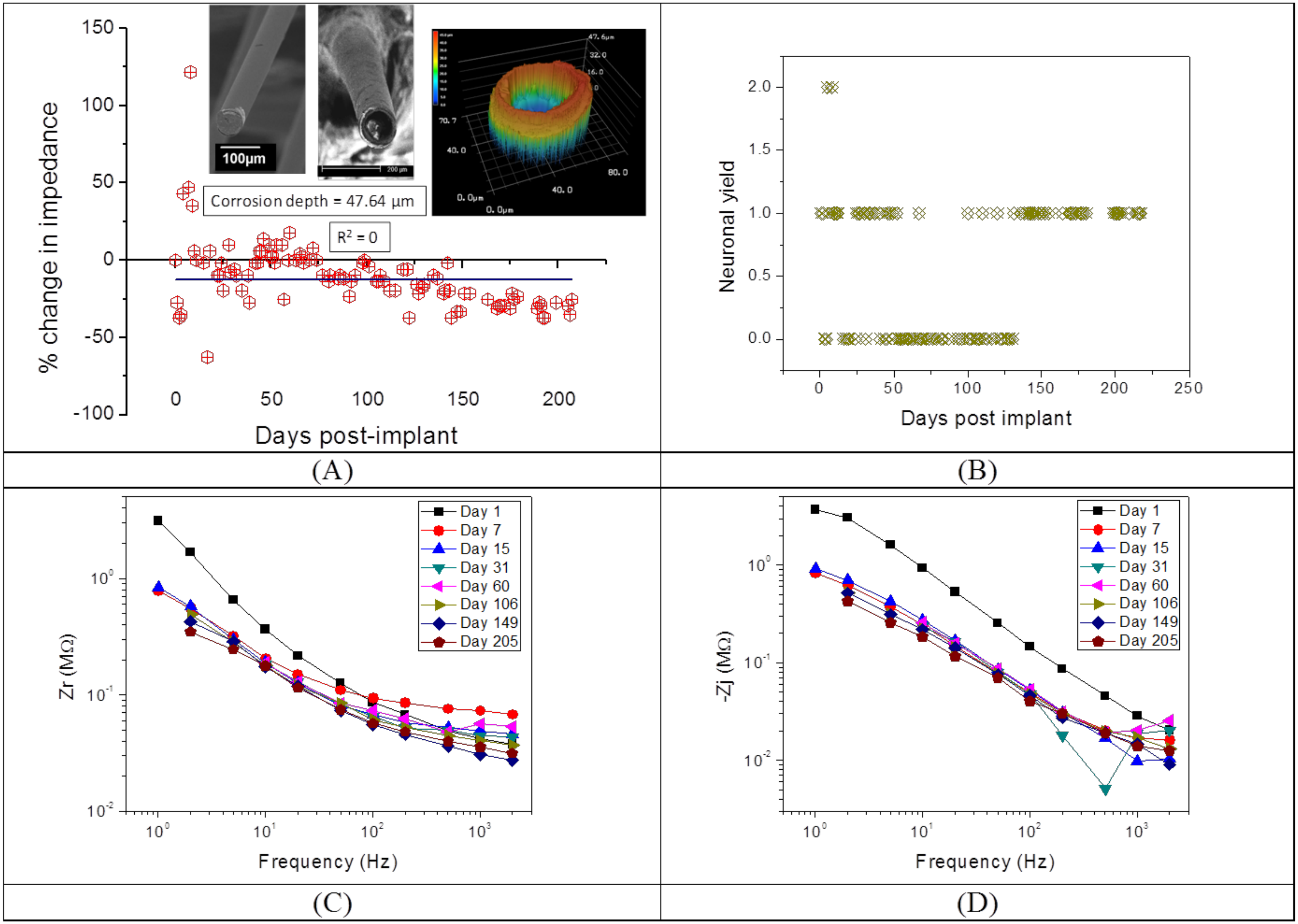

Figure 9: Wire 9 plots. A) Percentage change in in-vivo impedance plotted against the implanted duration and fitted with a Gaussian curve, B) Neuronal yield during the implanted period, C) Real part of the impedance across the measured frequency spectrum, and D) Imaginary part of the impedance across the measured frequency spectrum.

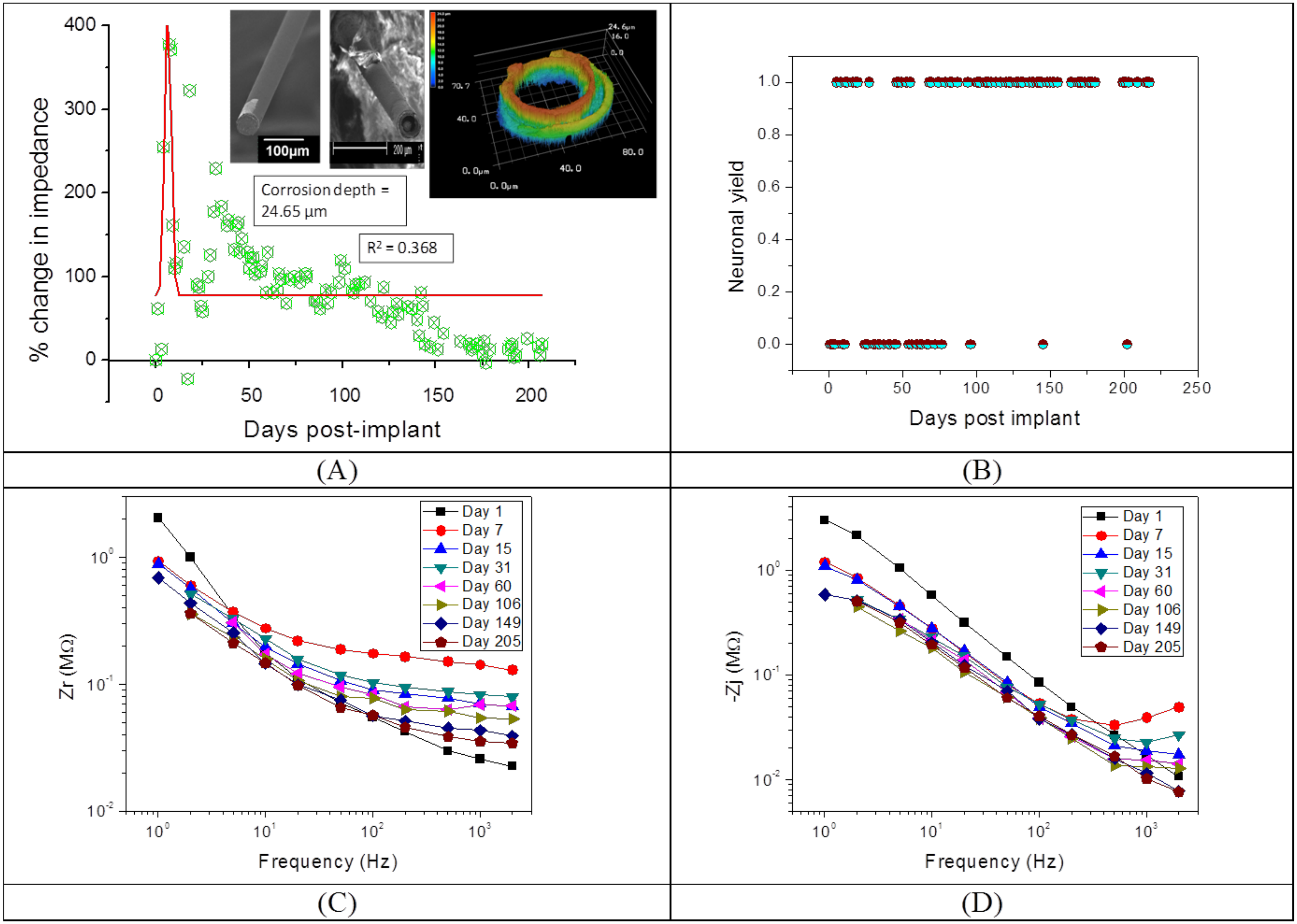

Figure 10: Wire 10 plots. A) Percentage change in in-vivo impedance plotted against the implanted duration and fitted with a Gaussian curve, B) Neuronal yield during the implanted period, C) Real part of the impedance across the measured frequency spectrum, and D) Imaginary part of the impedance across the measured frequency spectrum.

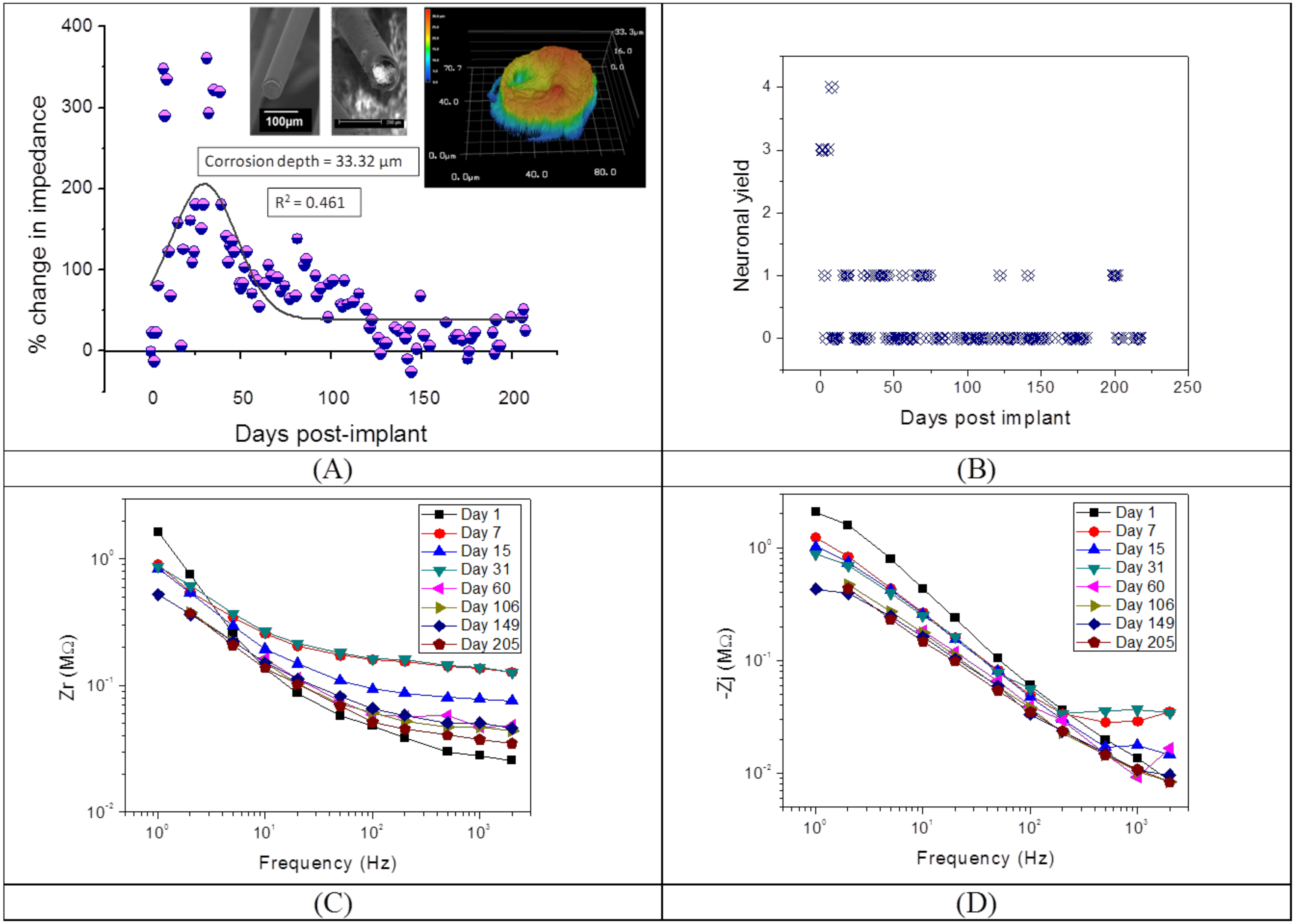

Figure 11: Wire 11 plots. A) Percentage change in in-vivo impedance plotted against the implanted duration and fitted with a Gaussian curve, B) Neuronal yield during the implanted period, C) Real part of the impedance across the measured frequency spectrum, and D) Imaginary part of the impedance across the measured frequency spectrum.

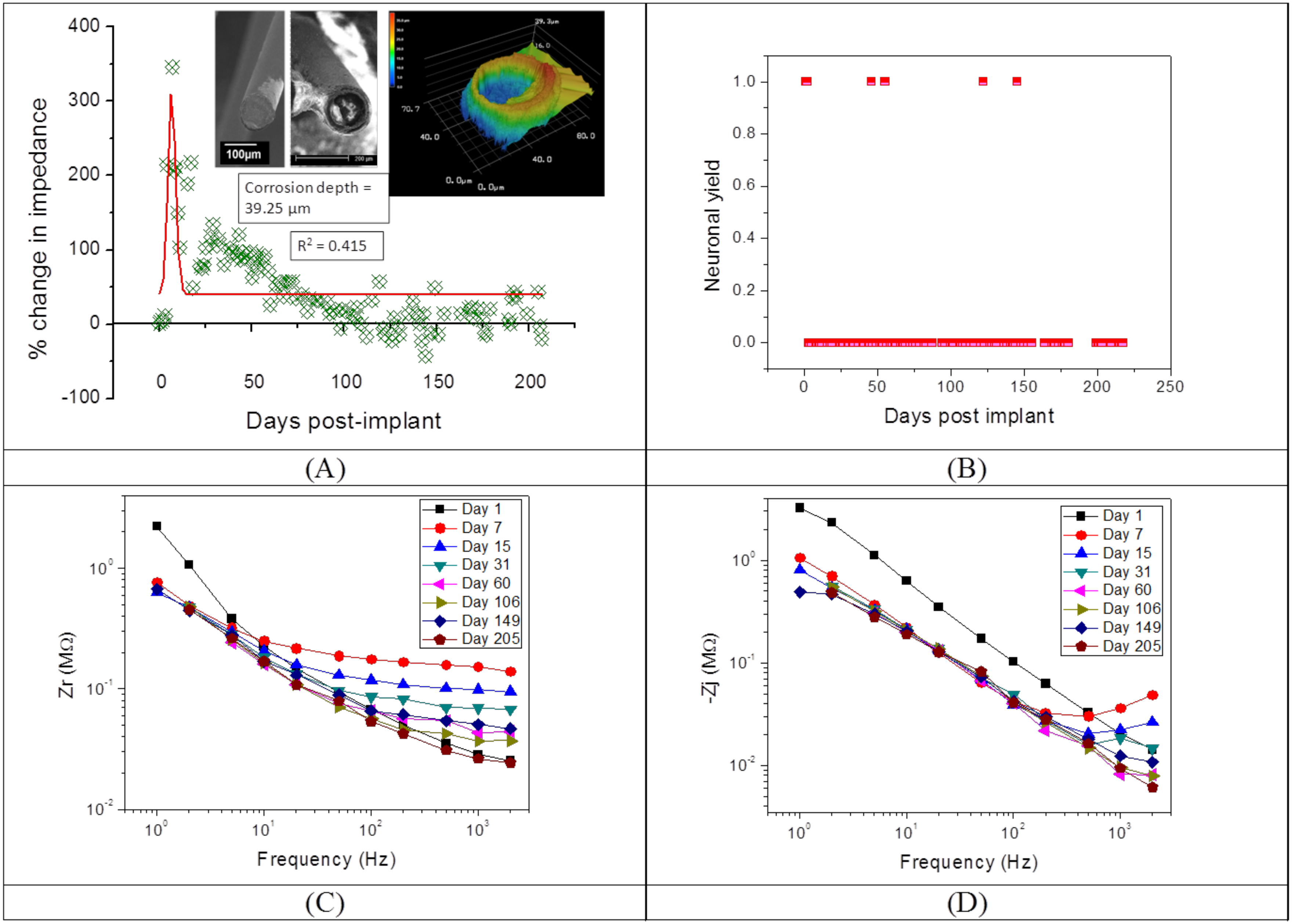

Figure 12: Wire 12 plots. A) Percentage change in in-vivo impedance plotted against the implanted duration and fitted with a Gaussian curve, B) Neuronal yield during the implanted period, C) Real part of the impedance across the measured frequency spectrum, and D) Imaginary part of the impedance across the measured frequency spectrum.
